# Loss of microbial diversity and pathogen domination of the gut microbiota in critically ill patients

**DOI:** 10.1099/mgen.0.000293

**Published:** 2019-09-17

**Authors:** Anuradha Ravi, Fenella D Halstead, Amy Bamford, Anna Casey, Nicholas M. Thomson, Willem van Schaik, Catherine Snelson, Robert Goulden, Ebenezer Foster-Nyarko, George M. Savva, Tony Whitehouse, Mark J. Pallen, Beryl A. Oppenheim

**Affiliations:** ^1^​ Quadram Institute Bioscience and University of East Anglia, Norwich, NR4 7UA, UK; ^2^​ NIHR Surgical Reconstruction and Microbiology Research Centre, Queen Elizabeth Hospital, Birmingham, B15 2GW, UK; ^3^​ Queen Elizabeth Hospital, University Hospitals Birmingham NHS Foundation Trust, Birmingham, B15 2GW, UK; ^4^​ Institute of Microbiology and Infection, University of Birmingham, Edgbaston, Birmingham B15 2TT, UK; ^5^​ McGill University, Montréal, QC H3G 2M1, Canada; ^6^​ School of Biological Sciences, University of East Anglia, Norwich Research Park, Norwich NR4 7TU, UK; ^7^​ School of Veterinary Medicine, University of Surrey, Daphne Jackson Rd, Guildford GU2 7AL, UK

**Keywords:** intensive care unit, microbiome, gut microbiota, pathogens, shotgun metagenomics, antimicrobial resistance, critical illness, meropenem

## Abstract

Among long-stay critically ill patients in the adult intensive care unit (ICU), there are often marked changes in the complexity of the gut microbiota. However, it remains unclear whether such patients might benefit from enhanced surveillance or from interventions targeting the gut microbiota or the pathogens therein. We therefore undertook a prospective observational study of 24 ICU patients, in which serial faecal samples were subjected to shotgun metagenomic sequencing, phylogenetic profiling and microbial genome analyses. Two-thirds of the patients experienced a marked drop in gut microbial diversity (to an inverse Simpson’s index of <4) at some stage during their stay in the ICU, often accompanied by the absence or loss of potentially beneficial bacteria. Intravenous administration of the broad-spectrum antimicrobial agent meropenem was significantly associated with loss of gut microbial diversity, but the administration of other antibiotics, including piperacillin/tazobactam, failed to trigger statistically detectable changes in microbial diversity. In three-quarters of ICU patients, we documented episodes of gut domination by pathogenic strains, with evidence of cryptic nosocomial transmission of *Enterococcus faecium *. In some patients, we also saw an increase in the relative abundance of apparent commensal organisms in the gut microbiome, including the archaeal species *Methanobrevibacter smithii *. In conclusion, we have documented a dramatic absence of microbial diversity and pathogen domination of the gut microbiota in a high proportion of critically ill patients using shotgun metagenomics.

## Data Summary

Metagenome sequences have been deposited in the Sequence Read Archive under Bioproject reference PRJNA533528: https://www.ncbi.nlm.nih.gov/bioproject/PRJNA533528

**Impact Statement**

While much work on the gut microbiota looks at subtle changes that might influence the balance between health and disease, here we show that the gut microbiota in the critically ill represents a worst-case scenario, where the usual rich and versatile microbial community of the gut is often replaced by a grossly simplified microbiota, dominated by drug-resistant pathogens. Our documentation of this worrying phenomenon in a prospective observational study establishes the extent of the problem in a uniquely vulnerable population, while also highlighting the potential of shotgun metagenomics as a powerful new tool in microbial surveillance. In achieving strain-level resolution of pathogen genomes from faecal metagenomes, we have uncovered cryptic transmission of nosocomial pathogens among intensive care unit patients. In documenting episodes of gut domination by pathogens and apparent commensals*,* our findings raise important questions about the potentially clinically relevant metabolic and physiological consequences of such events. The fact that not all intravenous antibiotics are equally disruptive of gut microbial ecology highlights the potential for the optimization of microbiome-sparing antibiotic regimes. More generally, our observations pave the way for precise patient-specific interventions that maintain or restore gut microbial diversity in the ICU, including enhanced infection control and tailored use of microbiota-sparing antibiotics, plus oral administration of antibiotic-absorbing charcoal or beta-lactamase.

## Introduction

Interest has recently focused on the gut microbiota in long-stay patients on the adult intensive care unit (ICU) [1–3]. Unfortunately, many life-saving measures applied to ICU patients potentially have negative impacts on the gut microbiota – examples include assisted ventilation, enteric feeds and a range of medications, including broad-spectrum antibiotics, proton pump inhibitors, inotropes and opioids [4–6]. In recent years, interest has grown in protecting or restoring the integrity of the gut microbiome in ICU patients, using ecological approaches such as probiotics or faecal microbiota transplants [7–18]. Similarly, surveillance of pathogens and of antimicrobial resistance in the gut of critically ill patients has potential benefit in predicting infection and guiding treatment or infection control measures [19–21]. However, in the absence of high-precision approaches to the surveillance of complex microbial communities, it remains unclear which ICU patients might benefit from interventions affecting the gut microbiota and how such interventions should be targeted for optimum effect.

Fortunately, recent advances in sequencing and bioinformatics have made shotgun metagenomics an attractive approach in precision medicine [22, 23]. We therefore undertook a prospective observational study of 24 ICU patients, in which serial faecal samples were subjected to shotgun metagenomic sequencing, phylogenetic profiling and microbial genome analyses, with the aims of evaluating the utility of shotgun metagenomics in long-stay ICU patients, documenting the dynamics of the gut microbiota in this context and determining how it is affected by relevant clinical and demographic factors.

## Methods

### Study design and human subjects

Queen Elizabeth Hospital Birmingham is a university teaching hospital serving a population of approximately 1.5 million with a wide range of tertiary services, including solid organ and bone marrow transplantation. Patients were enrolled for study participation if they were aged over 18 years and had been admitted to the ICU within the previous 72 h and were expected to remain there for more than 48 h. Patients were considered to be evaluable if their first stool sample and at least one subsequent sample were collected on the ICU.

Patient information was collected on a case report form, which included information on gender, age, reason for admission, severity of disease scores, length of hospital stays prior to ICU admission, current antibiotic therapy, blood markers, details of nutrition, drugs and relevant clinical microbiology results. The study started in May 2017 and ended in February 2018, when data and specimen collection for the 30th participant had been completed.

### Sample collection, storage and DNA extraction

The first faecal sample passed each calendar day by each enrolled patient on the ICU was collected and sent to the research team. Stool samples were aliquoted and then frozen at −20 °C as soon as possible after collection. They were then shipped frozen to the Quadram Institute in Norwich, where they were stored at −80 °C. Time and date of collection and transport was noted. Faecal samples were destroyed at the end of the study. Around 0.1 to 0.2 g of frozen faecal sample was used for DNA extraction. The extraction was carried out using the FastDNA SPIN Kit for Soil (MP Biomedicals, CA, USA) according to the manufacturer’s instructions, except that 100 µl rather than 50 µl of DES elution buffer was used in the final elution.

Samples from extra-intestinal sites were collected when indicated on clinical grounds and processed by the hospital’s clinical microbiology laboratory using standard diagnostic procedures.

### Shotgun metagenomic sequencing

The DNA concentration was normalized using Qubit 4 (Invitrogen, Thermo Fisher, MA, USA) and sequencing libraries were prepared using the Nextera XT library preparation kit (Illumina). The DNA was fragmented, tagged, cleaned and normalized according to the manufacturer’s recommendations. The quality of the final pooled library was evaluated using Agilent 2200 Tape Station (Agilent) and the concentration was measured using Qubit 4 (Invitrogen, Thermo Scientific, MA, USA). Libraries were sequenced in batches on a NextSeq 550 using a high-output flow cell delivering 150 bp paired-end reads. The libraries were sequenced to a sequencing depth of ∼2 Gbp per sample.

Reads from the sequencer were uploaded onto virtual machines provided by the Medical Research Council (MRC) Cloud Infrastructure for Microbial Bioinformatics (CLIMB) project using BaseMount [24, 25]. Initially, the sequences were assessed for quality using FastQC (version 0.11.5) and SeqKit with the parameter ‘stats’ [26, 27]. Quality filtering was performed using Trimmomatic (version 0.35) with default parameters [28]. Trimmomatic’s Illuminaclip function was used to remove Illumina adapters. Human sequences were removed by mapping reads towards the human genome, Hg19, using BowTie 2 version 2.3.4.1 [29]. SAMtools [30] view was used with parameters (-f 12 -F 256) to extract unmapped reads (forward and reverse) and reads that are not primary alignments respectively. BEDtools bamtofastq was used to convert resulting BAM to FASTQ files [31]. Then, these sequences were deposited in the Sequence Read Archive under reference PRJNA533528.

### Taxonomic profiling and statistical analysis

Forward and reverse paired reads were merged for each sample and fed as input to MetaPhlAn2 v2.7.7, which was used for taxonomic assignment of reads in each sample [32]. Metaphlan2 output was merged using the Python script merge_metaphlan_tables.py. A species-only abundance table (Table S1, available in the online version of this article) was created using Text Wrangler v5.5.2. Species that occurred only once and species with a relative abundance below 1 % in the whole dataset were discarded. This abundance data table, along with the metadata, was used for diversity analyses.

Alpha diversity was assessed using the inverse Simpson’s index calculated from the MetaPhlAn2 output using the vegan package (version 2.5–4) in R (version 3.5.2) [33]. Use of meropenem and piperacillin/tazobactam was coded individually because of their clinical importance and high use in our dataset, while all other antimicrobials were grouped together in a single variable ‘other antimicrobials’ for the final multivariable analysis. To account for the long-term effects of antibiotics on microbial diversity and the absence of data on when antibiotics were started, antibiotic use variables were coded at each sampling point into one of four levels: no use, starting use, ongoing use and historic use. Episodes were classified as ‘starting’ if the antibiotic was started on the same day the sample was taken; ‘ongoing’ if the antibiotic was still being administered on the day of a sample being taken; ‘historic’ if the antibiotic had been used prior to the date of sample collection but was no longer being administered.

Linear mixed models were used to estimate the fixed effects on alpha diversity of time in relation to ICU admission, antibiotic use, time of sample storage and health status measured by sequential organ failure assessment (SOFA) score, and age and sex of the patient. The nlme package (version 3.1–137) in R (version 3.5.2) was used to estimate all models [33, 34]. For the mixed effects regression model, data from 228 samples were included in the final analysis. Nine samples were excluded because the SOFA score was missing. The dataset included 42 samples where meropenem was administered and 44 where piperacillin/tazobactam was administered. Random patient-level effects on intercept and slope (linear change in diversity over time) were included in the model. An auto-regressive correlation structure (AR1) in discrete time was used to account for the residual autocorrelation due to longitudinal patient’s affect.

Subsampling of metagenome sequences was performed using Seqtk (https://github.com/lh3/seqtk) by adding the parameter ‘sample’. Metaphlan2 was rerun for the subsampled reads and the statistical analyses. Pairwise correlations between the diversity indices was analysed in Rstudio version 1.1.453.

### Metagenome-assembled genomes (MAGs)

For metagenomic binning, reads from each patient were co-assembled into contigs using MEGAHIT v1.1.3 [35]. Next, Anvi’o version 5.1 was used for mapping, binning, refining and visualizing the bins [36]. In brief, ‘anvi-gen-contigs-database’ was used with default settings to profile the contigs using Prodigal v2.6.3 and identify open reading frames [37]. Then, ‘anvi-run-hmms’ was used with default settings to identify bacterial, archaeal and fungal single copy gene collections using HMMER [38] and ‘anvi-run-cogs’ was used to predict gene functions in the contigs by using the National Center for Biotechnology Information’s (NCBI’s) Cluster of Orthologous Groups database. The taxonomy of the contigs was predicted using Centrifuge v1.0.3-beta [39] and added to the database using the ‘anvi-import-taxonomy-for-genes’ function. The reads from each sample of the respective patient were mapped to their corresponding co-assembled contigs using Bowtie 2 v2.3.4.1 and converted into sorted and indexed bam files using Samtools v1.9. Then, ‘anvi-profile’ was used to profile each bam file to estimate coverage and detection statistics for every contig in each sample. Next, ‘anvi-merge’ was used to combine the profiles of each sample and create a merged anvi’o profile. Then, ‘anvi-interactive’ was used to interactively visualize the distribution of the bins and identify MAGs.

We classified a genome bin as a MAG if it was more than 80 % complete and its redundancy was below 10 %. The completeness and redundancy for bacterial MAGs were assessed using ‘anvi-run-hmm’ with Anvi’o’s default HMM profiles associated with 139 single-copy genes [36]. Each bin was then refined using ‘anvi-refine’ based on tetranucleotide frequency, mean coverage, completion and redundancy. The program ‘anvi-summarize’ was used to generate an HTML output stat and FASTA file with the refined MAGs. To confirm completion and redundancy of the MAGs, CheckM v1.0.13 was used [40]. To recover MAGs for the fungal genomes, ‘anvi-run-hmms’ was used with BUSCO [41], a collection of 83 eukaryotic single-copy genes, while ‘anvi-compute-completeness’ was used to identify completion and ‘anvi-interactive’ was used to recover the MAGS.

For low-abundance pathogens that had been identified by MetaPhlAn2 but could not be recovered using Anvi’o, we constructed sets of completed taxon-specific reference genomes for each potential pathogen. Reference sequences were downloaded using the ‘ncbi-genome-download’ script [42]. We then mapped the metagenome from each sample against the relevant reference dataset using BowTie 2 version 2.3.4.1 [29]. The mapped reads were recovered using BEDtools bamtofastq and assembled into contigs using SPAdes (version 3.11.1) [43] and annotated using Prokka (version 1.12) [44]. Completion and contamination of these MAGs were assessed using CheckM. The coverage of the resulting draft genome sequences was calculated after mapping reads back to the assemblies using BowTie 2 and visualized with Qualimap2 [45]. To confirm species identity, average nucleotide identity was calculated from blast searches [46] or by using the online ANI/AAI matrix tool [47].

Resistance genes in the MAGs were identified using ABRicate v0.8.10 (https://github.com/tseemann/abricate) to find matches to resistance genes in the ResFinder database (consisting of 3021 sequences, updated on 20 October 2018) and the CARD database (consisting of 2237 sequences, updated on 20 October 2018) [42]. Default parameters were used for running ABRicate and the reports from the individual samples were compiled using the ‘—summary’ option. Only genes that had 100 % coverage to reference genes were only considered. *Candida albicans* MAGs were annotated using Prokka version 1.12 and the gene ERG11, encoding lanosterol 14-alpha demethylase, was extracted and searched for point mutations conferring resistance to fluconazole, itraconazole and/or voriconazole. For the identification of the mutations, blastn in the Mycology Antifungal Resistance Database (http://www.mardy.net/) was used.

For phylogenetic analyses of the MAGs, multi-locus sequence typing was performed using Torsten Seeman’s mlst program (https://github.com/tseemann/mlst). Complete *E. faecium* genomes of ST 80 were downloaded using the ‘ncbi-genome-download’ script (https://github.com/kblin/ncbi-genome-download). There were eight complete genomes in the taxonomy database. Core-genome single-nucleotide polymorphisms (SNPs) in the MAG and completed genomes from *E. faecium* were identified using Snippy v3.1 [29] and were then used to create a phylogeny with RAxML with a 100 rapid bootstrap analyses with the GTR-CAT model. The tree was rooted using the *E. faecium* DO complete genome (https://www.ncbi.nlm.nih.gov/genome/871?genome_assembly_id=169556). Genome comparisons between closely related MAGs were performed using blast Ring Image Generator [48].

### Pathogen culture

For the isolation of *Escherichia coli *, *C. albicans* and *E faecium.,* two separate aliquots (0.1–0.2 g) of each stool sample were loaded into 1.5 ml microcentrifuge tubes under aseptic conditions. One millilitre of physiological saline (0.85%) was added and the saline–stool samples were vortexed for 2 min at maximum speed to homogenize the samples completely. The homogenized samples were taken through eight 10-fold serial dilutions and 100 µl aliquots from each dilution were dispensed onto tryptone–bile–X–glucoronide agar, Sabouraud dextrose agar and Slanetz and Bartley medium (Oxoid). Both aliquots were plated in triplicate. The sample suspensions were spread on the plates using the cross-hatching method for confluent growth. Inoculated plates were incubated at 37 °C for 18–24 h (for tryptone–bile–X–glucoronide agar and Sabouraud dextrose agar) or for 48 h on Slanetz and Bartley medium.

Following incubation, the plates were examined for growth. On tryptone–bile–X–glucoronide agar, raised blue–green colonies with entire margins were taken as being indicative of the growth of *E. coli *. On Sabouraud dextrose agar, raised white-to-cream entire colonies with yeast-like appearance were scored as *Candida*. On Slanetz and Bartley medium, smooth pink-to-red colonies with a whitish margin were indicative of the growth of *Enterococcus *. Colonies were counted on the dilution plate that showed the highest number of discrete colonies and the colony count for each of the triplicate plates per dilution was recorded.

### High-throughput qPCR

Real-time quantitative PCR was performed using the LightCycler 480 (Roche, Germany) apparatus. Universal bacterial primers [43, 49] were used to determine the 16S rRNA copy number. Real-time PCR analyses were performed using LightCycler 480 SYBR Green 1 Master Mix (Roche, Germany) following the manufacturer’s instructions and at an annealing temperature of 56 °C. The DNA concentration was measured in a Qubit Broad Range assay kit (Invitrogen, Thermo Fisher, MA, USA) and the concentration was normalized to 5 ng µl^−1^. A 10 µl reaction was used with 0.1 mM of the universal primers. DNase-free water was used for negative controls. Standard curves were generated from *E. coli* standards normalized to 5 ng µl^−1^ and the copy numbers of the samples were calculated using standard curves. In order to assess the primer specificity, melt curve analyses were performed after qPCR using Fluidigm melting curve analysis software (http://fluidigm-melting-curve-analysis.software.informer.com/).

## Results

We initially recruited 30 serially recruited adult patients who were expected to stay on the ICU for >48 h. As is typical of ICU patients, the study population was heterogeneous, including patients with little or no previous medical history (e.g. suffering from trauma or intracranial haemorrhage), as well as individuals with complex and chronic clinical conditions and varying immune function. A set of 24 long-stay ICU patients who provided more than 5 samples was selected for further study (Table 1; Table S2).

**Table 1.** Clinical features and gut microbial ecology of ICU patients

**Patient**	**Age, sex**	**Clinical features**	Minimum ISI*	**Peak pathogens in gut and % abundance†**	**Clinical samples with same pathogen**
**2**	64, F	Subarachnoid haemorrhage	3.9	* K. pneumoniae *, 17 %	
**4**	75, M	Aortic aneurysm repair	1.5	* E. coli *, 80 %	
**8**	59, M	Subarachnoid haemorrhage	2.2	None	
**10**	55, M	Multiple trauma	5.3	None	
**24**	59, M	Drug-induced hepatitis	2.4	* E. coli *, 62 %	
**25**	46, M	Subarachnoid haemorrhage; alcoholic liver disease	1.0	* E. faecium *, 99 %; *E. coli *, 38 %	Urine
**29**	80, M	Subcapsular haematoma; liver cancer	6.2	* Enterococcus faecium *, 30 %	
**31**	43, M	Subarachnoid haemorrhage; alcoholism	3.1	* P. mirabilis *, 18 %	Sputum
**35**	49, M	Lung transplant	1.0	*C. albicans*, 82 %	
**36**	30, M	Multiple trauma	1.9	* E. coli *, 68 %	
**37**	47, M	Multiple trauma	3.1	None	
**38**	47, M	Insertion of left ventricular assist device	1.0	*C. albicans*, 77 %; *E. faecium *, 38 %; *E. cloacae *, 29 %	Sputum, blood
**41**	61, M	Oesophagectomy	1.5	* E. faecium *, 81 %	
**45**	63, M	Multiple trauma	5.2	None	
**46**	25, M	Chest infection	4.0	* E. faecalis *, 29 %; *E. coli *, 24 %	Urine
**47**	46, M	Subdural haemorrhage; hepatitis C; alcoholism	4.8	* E. coli *, 10 %	
**49**	65, F	Intracerebral haematoma	10.2	None	
**51**	78, M	ST-elevation myocardial infarction	1.2	* E. faecium *, 89 %	
**52**	54, F	Aortic valve repair; Marfan syndrome	2.1	* E. faecium *, 69 %	
**53**	40, F	Anaemia; end-stage renal disease	1.0	* E. faecium *, 99 %; *Klebsiella oxytoca *, 24 %	Urine
**54**	66, M	Alcoholic liver disease	2.4	* Enterococcus raffinosus *, 63 %; *E. faecium *, 44 %	
**55**	66, F	Subdural haemorrhage	3.6	* E. faecium *, 49 %	
**57**	84, M	Cardiac arrest; cardiomyopathy	5.6	None	
**59**	77, M	Subdural haemorrhage	4.8	* E. faecalis *, 18 %	

*, lowest microbial diversity in serial faecal samples from each patient, as reflected by inverse Simpson’s index.

†, peak relative abundance of potential pathogens in serial faecal samples from each patient.

To track the gut microbial dynamics of individual patients, we performed metagenomic sequencing of serial faecal samples (Table S1), followed by community analysis to determine the relative abundance of microbial species. The inverse Simpson’s index was calculated to assess microbial diversity (Table S3). Median time to receipt of a sample (where timings were available) from collection to storage was 2.6 h; 70 % of samples were received within 6 h and 87 % within 12 h. We found no association between changes in microbial diversity and time to receipt of sample or with the proportion of human reads in the sample (Table S4).

### Loss of gut microbial diversity and pathogen domination with meropenem

In two-thirds of patients, we saw a fall in diversity at some stage during their stay in ICU to an inverse Simpson’s index of <4 (Table 1; Table S3). An equivalent loss of microbial diversity was seen when sequence datasets were down-sampled to two million or to one million reads, showing that this is not the result of shallow or uneven sampling (Fig. S1). In a third of our patients, diversity fell, in at least one sample, to a precipitously low level, with an inverse Simpson’s index of <2, echoing findings from previous studies that used 16S rRNA gene amplicon sequencing [50, 51]. A fall in diversity was typically accompanied by domination in terms of the relative abundance of a single micro-organism in the sample.

**Fig. 1.**
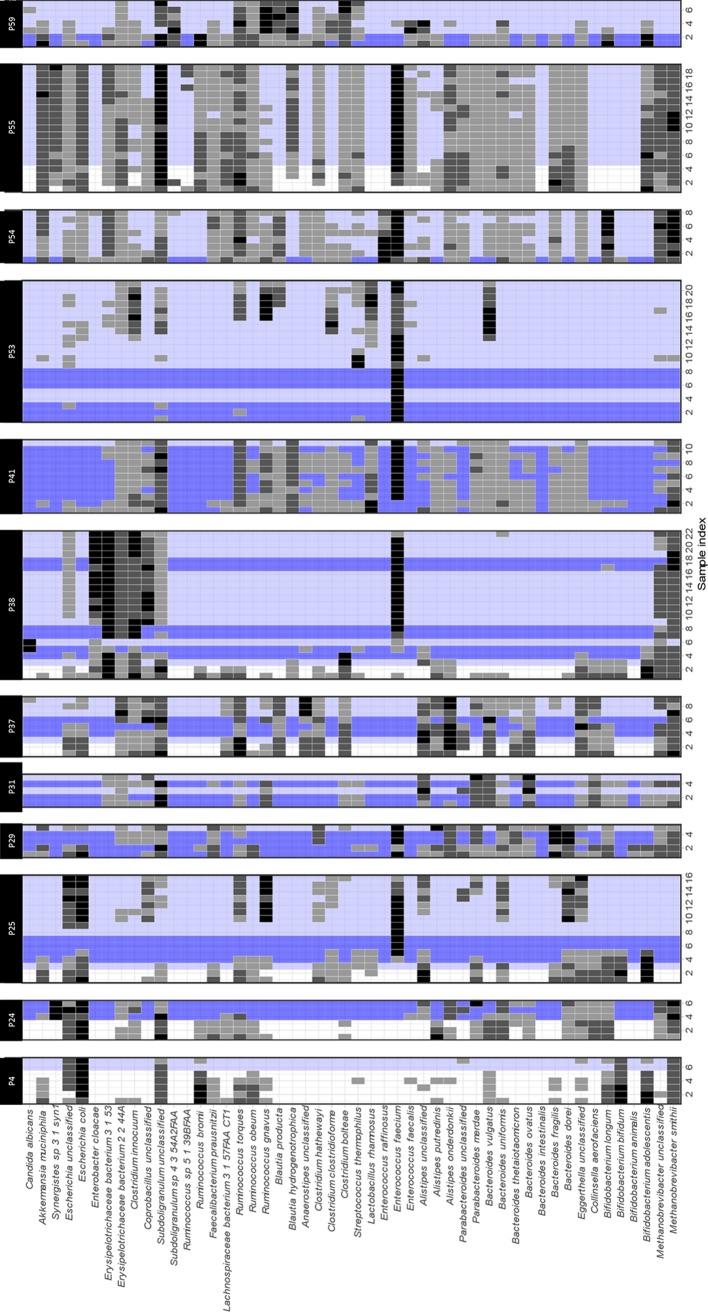
Pathogen domination of the gut microbiota. Timelines for patients showing pathogen domination, with relative abundance assessed by percentage of reads mapping to MAGs. Various antibiotics were given for treatment purposes during the study period.

In 75 % of the long-stay ICU patients, we saw a marked increase in the relative abundance of individual pathogens in stool samples. These included ESKAPE pathogens (*Enterococcus faecium, Klebsiella pneumoniae, Enterobacter cloacae*), other species of *Enterobacteriaceae* (* E. coli *, *Klebsiella oxytoca *, *Proteus mirabilis *) and the fungal pathogen *C. albicans*. During these episodes of pathogen domination, particularly for *E. faecium *, *E. coli* and *C. albicans*, the relative abundance of the pathogen often exceeded 50 % of sequence reads – in one patient, patient 53, in seven consecutive samples, >80 % of evaluable sequences were assigned to *E. faecium* (Fig. 1).

We found no statistically significant associations between microbial diversity and stool consistency (Bristol stool index) or SOFA score (Table 2). All but one of the ICU patients received antimicrobial chemotherapy at some point during their ICU stay, most commonly with the broad-spectrum agents piperacillin/tazobactam or meropenem. Surprisingly, piperacillin/tazobactam failed to trigger statistically detectable changes in microbial diversity, despite the apparent sensitivity of gut commensals to such agents [6]. However, current use of the intravenous agent meropenem was significantly associated with loss of gut microbial diversity and domination of pathogens in our ICU patients [change in inverse Simpsons index= −1.8, 95 % confidence interval (CI)= −3.4 to −0.25; *P*=0.024; Table 2). Similarly profound ecological changes have been reported in a study involving healthy volunteers given meropenem [52].

**Table 2.** Gut microbial diversity and clinical factor coefficients from a mixed effects regression model measuring the association between faecal microbial alpha diversity (inverse Simpson’s index) and demographics and clinical factors. Total of *n*=228 samples included in the analysis

	**Unit/level**	***n*/mean (sd**)	**Coefficient**	**95 % CI**	***P*-value**
Age	Per year	54.6 (14.8)	0.03	(−0.03, 0.09)	0.382
Sex	Male vs female	170	−0.14	(−2.40, 2.12)	0.897
Time since admission	Per day	18.1 (12.5)	−0.03	(−0.10, 0.04)	0.421
Meropenem	No use	114	0	Reference	
	Ongoing	42	−1.82	(−3.40, 0.25)	0.024*
	Starting	7	−1.30	(−3.03, 0.44)	0.143
	History	65	−1.29	(−2.92, 0.35)	0.122
Piperacillin/tazobactam	No use	51	0	Reference	
	Ongoing	44	0.66	(−1.09, 2.42)	0.456
	Starting	4	1.50	(−0.87, 3.87)	0.214
	History	129	0.83	(−0.92, 2.58)	0.350
Other antimicrobial	No use	32	0	Reference	
	Ongoing	55	−1.16	(−3.12, 0.79)	0.242
	Starting	8	−0.03	(−2.15, 2.09)	0.980
	History	133	−0.99	(−2.83, 0.85)	0.290
Bristol stool index	1–3	9	0	Reference	
	4	28	−0.54	(−2.25, 1.17)	0.536
	5	48	0.32	(−1.40, 2.04)	0.715
	6	75	−0.19	(−1.83, 1.46)	0.823
	7	62	−0.70	(−2.41, 1.02)	0.423
	Missing	6	0.04	(−2.22, 2.30)	0.975
SOFA score	Per point	6.1 (3.4)	−0.15	(−0.31, 0.01)	0.065

SOFA score, sequential organ failure score; higher values, greater morbidity.

*P<0.05.

*n*: number of samples with each level of covariate; sd: standard deviation.

### Impact on gut commensals

Antibiotics are known to provoke overgrowth in the gut of microbial species not known to be pathogens [52–54]. We saw the relative abundance of the archaeon *Methanobrevibacter smithii* exceed 10 % of reads in nine ICU patients. Quantitative PCR investigations, confirming that bacterial biomass did not change over time, suggest that this reflects an increase in abundance of this archaeon in real terms (Fig. S2), even though this organism always remained a minority component of the microbiota. Interestingly, recent publications have shown that the cultural and ecological requirements for *M. smithii* are far simpler than previously thought, supported even by individual species of bacterial pathogens or commensals [55–57] Furthermore, isolation of this organism from vaginal and urine samples raises the question of whether it should always be considered a harmless commensal [57, 58].

**Fig. 2.**
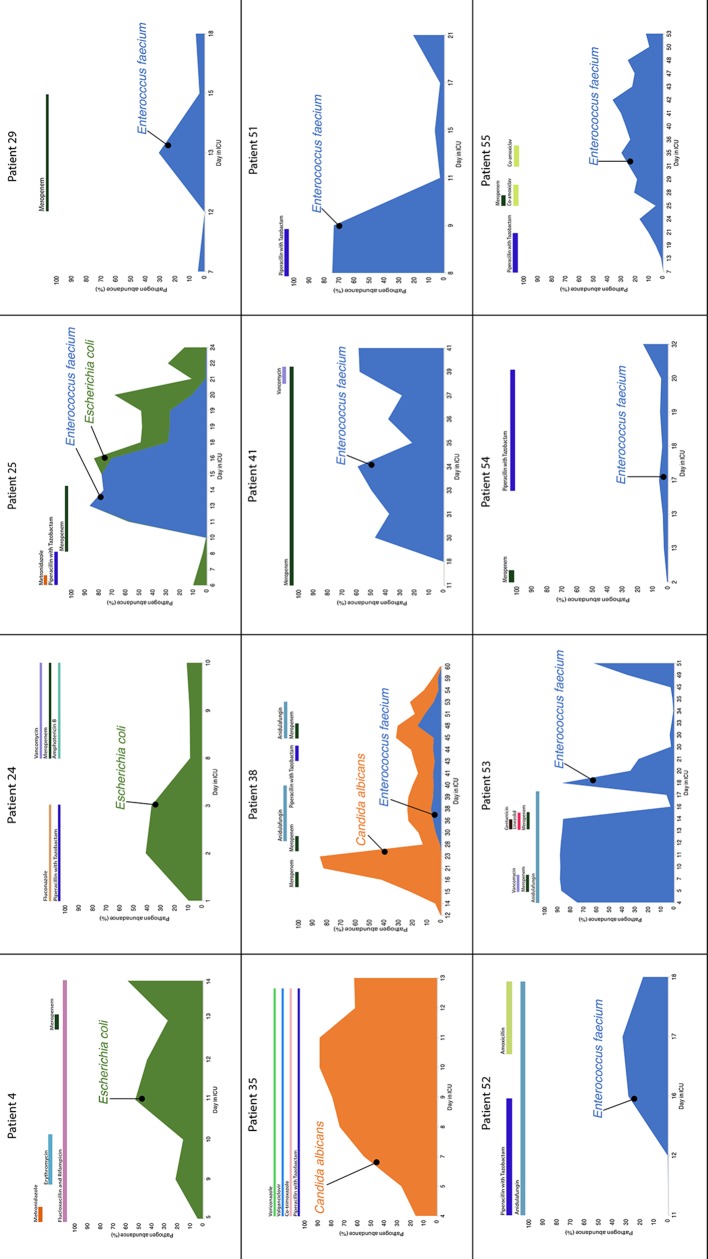
abundance of gut micro-organisms among patients who began meropenem during the study This heat map shows the top 50 taxa by average relative abundance across the whole dataset. Greyscale shading of cells shows relative abundance: 0, no shading; 0–1 %, light grey; 1–10 %, mid-grey; >10 %, dark grey. Coloured shading of columns reflects meropenem use: no use, blank; ongoing use, dark blue; history of meropenem, light blue.

Other apparent commensals showing rises in relative abundance to >50 % include *Streptococcus thermophilus *, *Alistipes onderdonkii *, *Bifidobacterium longum *, an unnamed species from *Erysipelotrichaceae, Ruminococcus torques* and an unclassified species of *Subdoligranulum* (Fig. 2; Table S1). We also observed reductions in the relative abundance of gut bacteria when comparing the sample with the highest diversity (typically first day in ICU) to the last sample for each patient (Table S5), with loss of *Ruminococcus gnavus, R torques., Faecalibacterium prausnitzii and Colinsella aerofaciens* evident in at least five patients.

### MAGs and nosocomial transmission of pathogens

We obtained MAGs of the potential pathogens and used them to reconstruct pathogen biology and epidemiology, including multi-locus sequence types (Table S6). We found that pathogen blooms within an individual patient were typically clonal, i.e. caused by a single strain. Pathogens dominating the gut microbiota in ICU patients were also typically inherently resistant to antibiotics or possessed genes associated with antimicrobial resistance – vancomycin-resistance genes were detected in two strains of *E. faecium* and aminoglycoside resistance genes in two strains of *E. coli *, one of which also encoded an extended-spectrum beta-lactamase (Table S7). Metagenomic surveys of resistance genes in samples typically reported profiles almost completely matching the resistance genes present in the dominating pathogen. However, we did detect in some samples genes associated with resistance to macrolides (*mph*A and *msr*C genes) and aminoglycosides (*ant* and *aph* genes) that were absent from any of our MAGs (Fig. S3).

Enterococcal blooms were seen in 11 patients. In six cases, the dominant strain belonged not just to the same species*, E. faecium*, but also to the same sequence type, ST80, which is a well-documented cause of nosocomial outbreaks across the globe [59–61]. Core phylogenetic analyses with the MAGs identified in this study and complete public *E. faecium* ST 80 genomes from the NCBI database indicated that the *E. faecium* MAGs of patients 51, 54, 55 were closely related MAGs. The *E. faecium* MAGS of patients 51 and 54 differed by 39 SNPs and those of patients 51 and 55 differed by 142 SNPs in their core genomes (Fig. S4). Interestingly, all three patients had overlapping stays in adjacent rooms on the ICU.

### Quantification of pathogens

In several patients (patients 4, 24, 25, 41, 51, 53 and 55), we found that an increase in the relative abundance of sequences assigned to a bacterial pathogen (* E. coli* or *E. faecium *) occurred in association with an increase in total bacterial biomass (determined by qPCR) and/or an increase in pathogen abundance (determined by quantitative culture), confirming that pathogen abundance increased in absolute as well as in relative terms (Fig. S5). However, similar increases in the relative abundance of the fungal pathogen *C. albicans* in patients 35 and 38 were accompanied by a decrease in bacterial 16S rRNA copy number and a lack of increase in abundance on quantitative culture, suggesting that that apparent changes in the abundance of this fungal pathogen are best explained by loss of bacterial biomass.

## Discussion

Here, we have shown the utility of applying shotgun metagenomics to ICU patients for surveillance of the gut microbiota, documenting the loss of gut microbial diversity and domination of the gut by drug-resistant pathogens. Our use of shotgun metagenomics confirms the results of previous studies on ICU patients using less powerful sequence-based approaches, linking loss of gut microbial diversity to adverse clinical outcomes and loss of colonization resistance [19–21, 50, 51, 62–69]. However, with shotgun metagenomics, we have been able to reconstruct informative metagenome-assembled genomes, allowing us to characterize pathogens, identify resistance determinants and document cryptic nosocomial transmission of a clone of *E. faecium* that colonized three patients.

It is well established that administration of antibiotics leads to loss of diversity in the gut microbiota [70–73]. Nonetheless, although all but one of our patients received antibiotics, we only saw a statistically significant loss of diversity – and marked loss of beneficial organisms – after the administration of meropenem. Similar profound and longstanding effects of this agent on gut microbial diversity have been documented in healthy adults [52]. Although we were unable to detect any effect of other antimicrobials, given the small sample size, we cannot rule out small but significant effects for less commonly used agents.

The contrast between the effect of meropenem and the apparent lack of effect of other broad-spectrum agents such as piperacillin/tazobactam suggests that pharmacokinetics plays a key role in determining impact on the gut microbiota and that there is scope for tailoring antibiotic regimes to spare the gut microbiota, building on previous studies confirming the low-risk status of ureidopenicillins such as piperacillin on the risk of *Clostridioides difficile* infection or colonization with vancomycin-resistant enterococci [74, 75].

We have used shotgun metagenomics to document domination of the gut microbiota by microbial pathogens in most ICU patients. Although from sequences alone, it is hard to determine whether increases in the relative abundance of pathogens reflect an increase in the biomass of pathogens or simply a loss of commensals [76, 77], we were able to use qPCR and microbial culture to confirm that, at least in some cases, there was a genuine increase in the absolute abundance of the pathogen.

In a quarter of our patients, in line with other similar attempts at sequence-based surveillance in vulnerable patients [19, 20], the same species of pathogen was isolated from clinical samples from outside the gut. However, as our clinical isolates were not subjected to genome sequencing, we cannot be certain that they belonged to the strains associated with domination of the gut.

Interestingly, we also saw episodes of ecological domination by apparent commensals. The significance of these episodes remains uncertain. A recent study has suggested that commensal bacteria carry diverse uncharacterized resistance genes that contribute to their selection after antibiotic therapy [78]. It is worth noting that *M. smithii *, like other archaea, is intrinsically resistant to antibiotics as a result of its distinctive non-bacterial biology [79].

We must acknowledge some limitations of this study: the sensitivity of metagenomics as a diagnostic remains uncertain and is unlikely to compete with culture – in terms of costs or sensitivity – in the detection and characterization of culturable pathogens present in low abundance. In addition, in its simplest form, the ability of shotgun metagenomics to link mobile elements to the chromosomes from their host cells is poor, although proximity linkage approaches might overcome this limitation [80].

## Conclusions

Here, we have shown that surveillance of the gut microbiota in long-stay ICU patients using shotgun metagenomics is capable of detecting episodes of low diversity and pathogen domination, as well as providing genome-level resolution of colonizing pathogens and evidence of cryptic nosocomial transmission. We have also shown that use of meropenem is associated with ecological disruption of the gut microbiota. These observations pave the way for precise patient-specific interventions that protect the gut microbiota (e.g. enhanced infection control, tailored use of microbiota-sparing antibiotics, oral administration of antibiotic-absorbing charcoal or of a beta-lactamase) [81, 82].

Although we failed to find a link between gut microbial diversity or pathogen domination of the gut and clinical outcomes in our group of ICU patients, such evidence has been documented for similar groups of vulnerable patients [19, 21, 63], where ecological approaches to restoring gut microbial diversity, such as faecal microbiota transplants, are under evaluation [16–18, 83, 84]. Similar intervention studies – underpinned by the kind of metagenomic surveillance we have established here – are likely to clarify whether maintenance or restoration of gut microbial diversity influences clinical outcomes in long-stay ICU patients.

## Data bibliography

1. Ravi, A. NCBI Bioproject PRJNA533528 (2019).

## Supplementary Data

Supplementary File 1

Supplementary File 2
