# Supplementary material for: Loss of microbial diversity and pathogen domination of the gut microbiota in critically ill patients
Source: Microb Genom. 2019 Sep 17;5(9):e000293. doi: 10.1099/mgen.0.000293 (PMC6807385; doi:10.1099/mgen.0.000293)
Supplement: Supplementary File 1 [file mgen-5-293-s001.pdf]

Supplementary Files

Table S1 MetaPhlAn2 species relative abundance output: see Excel file

Table S2. Patient data

Table S3. Diversity indices for all patients: see Excel file

Table S4. Proportion of human reads in the samples: see Excel file

Table S5. Loss of taxa

Table S6. Metagenome-assembled genomes

Table S7. Resistance profiles of MAGs

Figure S1. Sub-sampling of reads does not influence inverse Simpson index

Figure S2. Relative abundance of *Methanobrevibacter* and qPCR

Figure S3. Antibiotic resistance genes detected from metagenome reads

Figure S4. Phylogeny of the *Enterococcus faecium* MAGs and complete genomes

Figure S5: Abundance of pathogens by read mapping, qPCR and culture

ID					LENGTH OF STAY IN THE HOSPITAL	OF STAY IN THE ICU	MORTALITY	SAMPLES FOR SEQUENCING
**2**	64	F	Subarachnoid haemorrhage	COPD[1]	29	15	No	12
**4**	75	M	Aortic Aneurysm repair	COPD[1]	Transfer from another hospital	14	Yes	7
**8**	59	M	Subarachnoid haemorrhage	Nil	52	17	No	9
**10**	55	M	Multiple trauma	COPD[1]	0	13	No	5
**15**	56	M	CABG[2]	NIDDM[3]; Hypertension, endocarditis, Aortic valve stenosis	13	5	No	2
**22**	72	M	Pneumonia	TB[4]; NIDDM[3]	70	6	No	2
**24**	59	M	Drug induced hepatitis	Nil	23	17	Unknown	6
**25**	46	M	Intracerebral bleed	Hydrocephalus, alcoholic liver disease, intracerebral haemorrhage	29	24	Unknown	16
**29**	80	M	Subcapsular haematoma	Liver cancer	16	18	No	5
**31**	43	M	Subarachnoid haemorrhage	Hypertension; alcoholism	18	19	Yes	5
**35**	59	M	Lung transplant	COPD[1]	41	14	No	9
**36**	30	M	Multiple trauma		50	20	No	8
**37**	47	M	Multiple trauma	Depression	62	27	No	9
**38**	47	M	Insertion of left ventricular assist device	NIDDM[3], essential hypertension	133	60	No	22
**41**	41	M	Oesophagostomy	Oesophageal adenocarcinoma	144	45	No	11
**45**	63	M	Multiple trauma		51	26	No	11
**46**	25	M	Bacterial pneumonia	Nil	44	37	Yes	17
**47**	46	M	Acute subdural haematoma	Hepatitis C and schizophrenia	Unknown	20	No	7
**49**	65	F	Intracerebral haematoma	Breast cancer	28	11	No	6
**51**	78	M	ST-elevation myocardial infarction	Nil	37	27	No	6
**52**	54	F	Aortic surgery	Suspected endocarditis	59	20	No	5
**53**	40	F	Anaemia	End stage renal disease	94	42	No	21
**54**	66	M	Alcohol withdrawal syndrome	Epilepsy	47	32	Yes	8
**55**	66	F	Subdural haemorrhage	NIDDM[3]	56	55	No	19
**57**	84	M	Cardiac arrest	Hypertension; Cardiomyopathy	108	14	No	5
**59**	77	M	Subdural haematoma	Hyperlipidaemia; Hypertension	56	28	No	7

Table S2

Patient metadata data consisting of previous medical conditions and current medical complaints.

[1]Chronic Obstructive Pulmonary Disease; [2] Coronary Artery Bypass Grafting; [3]Non-Insulin Dependent Diabetes Mellitus; [4]Tuberculosis

Table S5

a) Number of species lost for each patient

Patient name	Highest diversity	Last day of sampling	Number of species lost
Patient 10	Day 6	Day 10	4
Patient 2	Day 1	Day 15	4
Patient 24	Day 1	Day 10	14
Patient 25	Day 6	Day 24	9
Patient 29	Day 7	Day 18	3
Patient 31	Day 12	Day 16	4
Patient 35	Day 4	Day 13	25
Patient 36	Day 11	Day 18	6
Patient 37	Day 10	Day 27	6
Patient 38	Day 14	Day 54	6
Patient 4	Day 5	Day 14	8
Patient 41	Day 11	Day 41	11
Patient 45	Day 6	Day 24	7
Patient 46	Day 12	Day 35	3
Patient 47	Day 6	Day 15	0
Patient 49	Day 7	Day 12	2
Patient 51	Day 9	Day 21	2
Patient 52	Day 11	Day 18	2
Patient 53	Day 35	Day 51	2
Patient 54	Day 2	Day 32	4
Patient 55	Day 7	Day 53	2
Patient 57	Day 4	Day 18	4
Patient 8	Day 4	Day 16	5

b) Patients and absence of species

**Microbial species**	**Number of patients**
Ruminococcus obeum	6
Collinsella_aerofaciens	5
Faecalibacterium_prausnitzii	5
Ruminococcus_gnavus	5
Alistipes putredinis	4
Bifidobacterium_adolescentis	4
*Coprobacillus*_unclassified	4
*Lachnospiraceae*_unclassified	4
Klebsiella_oxytoca	4
Clostridium_bolteae	4
Dorea_longicatena	4
*Methanobrevibacter*_unclassified	4
Alistipes_shahii	3
Alistipes unclassified	3
Bifidobacterium_bifidum	3
Bifidobacterium_longum	3
Escherichia_coli	3
Barnesiella_intestinihominis	3
*Subdoligranulum*_unclassified	3
Clostridium_clostridioforme	3
Coprococcus_comes	3
Ruminococcus torques	3
Alistipes_onderdonkii	2
Streptococcus_salivarius	2
Akkermansia_muciniphila	2
Anaerostipes_hadrus	2
Blautia_hydrogenotrophica	2
*Oscillibacter*_unclassified	2
Veillonella_parvula	2
Bacteroides vulgatus	2
Bacteroides_stercoris	2
Parabacteroides_merdae	2
*Parabacteroides*_unclassified	2
Clostridium_asparagiforme	2
Eubacterium_rectale	2
Ruminococcus_bromii	2
Streptococcus_anginosus	1
Streptococcus_mutans	1
Streptococcus_sanguinis	1
Streptococcus_vestibularis	1
Lactobacillus_gasseri	1
Lactobacillus_plantarum	1
Lactobacillus_reuteri	1
Bacteroides_dorei	1
Bacteroides_uniformis	1
Bacteroides_vulgatus	1
Clostridium_hathewayi	1
Clostridium_innocuum	1

Clostridium_leptum	1
Clostridium_nexile	1
Eubacterium_biforme	1
Eubacterium_cylindroides	1
Eubacterium_eligens	1
Eubacterium_hallii	1
Eubacterium_siraeum	1
Ruminococcus lactaris	1

Table S6

PATIENT NAME	MAG IDENTIFIED	REFERENCE SIZE (MB)	COMPLETENESS (%)	CONTAMINATION (%)	MLST
**PATIENT25**	*E. faecium*	2,987,425	99.63	0.87	787
**PATIENT29**	*E. faecium*	2,769,265	98.88	0.06	-
**PATIENT38**	*E. faecium*	2,689,224	99.63	0	262
**PATIENT41**	*E. faecium*	2,944,903	99.63	0.5	80
**PATIENT51**	*E. faecium*	2,908,317	99.63	0.56	80
**PATIENT52**	*E. faecium*	2,843,541	98.88	0	80
**PATIENT53**	*E. faecium*	2,837,393	99.25	0.12	80
**PATIENT54**	*E. faecium*	2,421,346	99.3	0.06	80
**PATIENT55**	*E. faecium*	2,907,337	99.44	0.56	80
**PATIENT4**	*E. coli*	4,015,777	76.47	2.29	-
**PATIENT25**	*E. coli*	4,970,123	98.65	1.24	315
**PATIENT36**	*E. coli*	4,288,275	97.57	0.11	538
**PATIENT24**	*E. coli*	4,646,804	83.17	2.09	-
**PATIENT35**	*C. albicans*	16116472	83.13	14.1	-
**PATIENT38**	*C. albicans*	15296317	76	7.2	-
**PATIENT 31**	*P. mirabilis*	3,819,203	100	0	
**PATIENT2**	*K. pneumoniae*	4,782,063	87.58	8.9	
**PATIENT38**	*Enterobacter sp.*	4,884,100	99.9	0.4	

Table S7

Identification of antibiotic resistance genes in the metagenome assembled genomes (MAGs)

***Enterococcus faecium* MAGs**

PATIENT NAME	PATHOGEN MAG	AMINOGLYCOSIDES	MACROLIDES AND STREPTOGRAMIN B	GLYCOPEPTIDES			
Antibiotic resistance genes[1]		*Aph*(3)	*Msr*C	*Van*A	*Van*H	*Van*X	*Van*Z
PATIENT 25	*Enterococcus faecium*	100	99	100	100	100	
PATIENT 29		99	100				
PATIENT 38			99				
PATIENT 41			99				
PATIENT 51			99				
PATIENT 52		100	99				
PATIENT 53		100	99	100	100	100	100
PATIENT 54		99	99				
PATIENT 55			99				

[1]Resistance genes were identified from ResFinder database. The numbers represent percentage of identity between the reference gene and query. Only query genes with 100% coverage with the reference genes were considered.

***Escherichia coli* MAGs**

PATIENT NAME	PATHOGEN MAG	AMINOGLYCOSIDES		MACROLIDES AND STREPTOGRAMIN B			BETALACTAM	
Antibiotic resistance genes[1]		*Aac*(3)	*Ant*(3)	*mef*B	*mph*A	*mdf*A	*bla*CTX	*bla*TEM
PATIENT 4	*Escherichia coli*	99.9		99.6	99.7	99.8		
PATIENT 25		99.8	99.3		99.6	98.2	100	100
PATIENT 36						98		
PATIENT 24						98.3		

[1]Resistance genes were identified from ResFinder database. The numbers represent percentage of identity between the reference gene and query. Only query genes with 100% coverage with the reference genes were considered.

Other MAGS

PATIENT NAME	PATHOGEN MAG	BETA-LACTAMS		QUINOLONES		ANTIFUNGAL AGENTS
Antibiotic resistance genes[1]		*bla*SHV	*bla*ACT	*oqx*A	*oqx*B	Fluconazole (ERG11)
PATIENT 2	*Klebsiella pneumoniae*	99.65		100	99.3	
PATIENT 38	*Enterobacter sp.*		99.83			
PATIENT 38	*Candida albicans*					99.43
PATIENT 35	*Candida albicans*					99.37

[1]Resistance genes for bacteria were identified from ResFinder database. The numbers represent percentage of identity between the reference gene and query. Only query genes with 100% coverage with the reference genes were considered. Resistance genes for fungi were identified using MARDy database and the numbers represent percentage of identity with 100% coverage of query and reference gene.

More info on ERG11 gene point mutations

BLASTn result downloaded from MARDy

Patient	Organism	Gene name	AA mutation	E value	%identity	Drug	Reference
38	*Candida albicans*	ERG111	Y132F Y205E Y257H D116E K143Q	0	99.8	Fluconazole	10.1111/1567-1364.12042
35	*Candida albicans*	ERG111	Y132F Y205E Y257H D116E K143Q	0	99.8	Fluconazole	10.1111/1567-1364.12042

ERG11 gene sequence

>Patient35 Lanosterol 14-alpha demethylase
ATGGCTATTGTTGAAACTGTCATTGATGGCATTAATTATTTTTTGTCCCTTAGTGTTACA
CAACAGATCAGTATATTATTAGGGGTTCCATTTGTTTACAACTTAGTATGGCAATATTTA
TATTCATTAAGAAAAGATAGAGCTCCATTAGTGTTTTATTGGATTCCTTGGTTTGGTTCT
GCAGCTTCATATGGTCAACAACCTTATGAATTTTTCGAATCATGTCGTCAAAAGTATGGT
GATGTATTTTCATTTATGTTATTAGGGAAAATTATGACGGTTTATTTAGGTCCAAAAGGT
CATGAATTTGTTTTCAATGCTAAATTATCTGATGTTTCTGCTGAAGAAGCTTATAAGCAT
TTAACTACTCCAGTTTTCGGTACAGGGGTTATTTATGATTGTCCAAATTCTAGATTAATG
GAACAAAAAAAATTTGCTAAATTTGCTTTGACTACTGATTCATTTAAAAGATATGTTCCT
AAGATTAGAGAAGAAATTTTGAATTATTTTGTTACTGATGAAAGTTTCAAATTGAAAGAA
AAAACTCATGGGGTTGCCAATGTTATGAAAACTCAACCAGAAATTACTATTTTCACTGCT
TCAAGATCTTTATTTGGTGATGAAATGAGAAGAATTTTTGACCGTTCATTTGCTCAATTA
TATTCTGATTTAGATAAAGGTTTTACCCCTATTAATTTTGTTTTCCCTAATTTACCTTTA
CCTCATTATTGGAGACGTGATGCTGCTCAAAAGAAAATCTCTGCTACTTATATGAAAGAA
ATTAAACTGAGAAGAGAACGTGGTGATATTGATCCAAATCGTGATTTAATTGATTCCTTA
TTGATTCATTCAACTTATAAAGATGGTGTGAAAATGACTGATCAAGAAATTGCTAATCTT
TTAATTGGTATTCTTATGGGTGGTCAACATACTTCTGCTTCTACTTCTGCTTGGTTCTTG
TTACATTTAGGTGAAAAACCTCATTTACAAGATGTTATTTATCAAGAAGTTGTTGAATTG
TTGAAAGAAAAAGGTGGTGATTTGAATGATTTGACTTATGAAGATTTACAAAAATTACCA
TCAGTCAATAACACTATTAAGGAAACTCTTAGAATGCATATGCCATTACATTCTATTTTT
AGAAAAGTTACTAACCCATTAAGAATCCCTGAAACCAATTATATTGTTCCAAAAGGTCAT
TATGTTTTAGTTTCTCCAGGTTATGCTCATACTAGTGAAAGATATTTTGATAACCCTGAA
GATTTTGATCCAACTAGATGGGATACTGCTGCTGCCAAAGCTAATTCTGTTTCATTTAAC
TCTTCTGATGAAGTTGATTATGGGTTTGGGAAAGTTTCTAAAGGGGTTTCTTCACCTTAT
TTACCATTTGGTGGTGGTAGACATAGATGTATTGGGGAACAATTTGCTTATGTTCAATTG
GGAACCATTTTAACTACTTTTGTTTATAACTTAAGATGGACTATTGATGGTTATAAAGTG
CCTGACCCTGATTATAGTTCAATGGTGGTTTTACCTACTGAACCAGCAGAAATCATTTGG
GAAAAAAGAGAAACTTGTATGTTTTAA

>Patient 38 Lanosterol 14-alpha demethylase
ATGGCTATTGTTGAAACTGTCATTGATGGCATTAATTATTTTTTGTCCCTTAGTGTTACA
CAACAGATCAGTATATTATTAGGGGTTCCATTTGTTTACAACTTAGTATGGCAATATTTA
TATTCATTAAGAAAAGATAGAGCTCCATTAGTGTTTTATTGGATTCCTTGGTTTGGTTCT
GCAGCTTCATATGGTCAACAACCTTATGAATTTTTCGAATCATGTCGTCAAAAGTATGGT
GATGTATTTTCATTTATGTTATTAGGGAAAATTATGACGGTTTATTTAGGTCCAAAAGGT
CATGAATTTGTTTTTAATGCTAAATTATCTGATGTTTCTGCTGAAGATGCTTATAAACAT
TTAACTACTCCAGTTTTCGGTAAAGGGGTTATTTATGATTGTCCAAATTCCAGATTAATG
GAACAAAAAAAATTTGCTAAATTTGCTTTGACTACTGATTCATTTAAAAGATATGTTCCT
AAGATTAGAGAAGAAATTTTGAATTATTTTGTTACTGATGAAAGTTTCAAATTGAAAGAA
AAAACTCATGGGGTTGCCAATGTTATGAAAACTCAACCAGAAATTACTATTTTCACTGCT
TCAAGATCTTTATTTGGTGATGAAATGAGAAGAATTTTTGACCGTTCATTTGCTCAACTA
TATTCTGATTTAGATAAAGGTTTTACCCCTATTAATTTTGTTTTCCCTAATTTACCTTTA
CCTCATTATTGGAGACGTGATGCTGCTCAAAAGAAAATCTCTGCTACTTATATGAAAGAA
ATTAAACTGAGAAGAGAACGTGGTGATATTGATCCAAATCGTGATTTAATTGATTCCTTA
TTGATTCATTCAACTTATAAAGATGGTGTGAAAATGACTGATCAAGAAATTGCTAATCTT
TTAATTGGTATTCTTATGGGTGGTCAACATACTTCTGCTTCTACTTCTGCTTGGTTCTTG
TTACATTTAGGTGAAAAACCTCATTTACAAGATGTCATTTATCAAGAAGTTGTTGAATTA
TTGAAAGAAAAAGGTGGTGATTTGAATGATTTGACTTATGAAGATTTACAAAAATTACCA
TCGGTCAATAACACTATTAAGGAAACTCTTAGAATGCATATGCCATTACATTCTATTTTC
AGAAAAGTTACTAACCCATTAAGAATCCCTGAAACCAATTATATTGTTCCAAAAGGTCAT
TACGTTTTAGTTTCTCCAGGTTATGCTCATACTAGTGAAAGATATTTTGATAACCCCGAA
GATTTTGATCCAACTAGATGGGACACTGCTGCTGCCAAAGCTAATTCTGTTTCATTTAAC
TCTTCTGATGAAGTTGATTATGGGTTTGGGAAAGTTTCTAAAGGGGTTTCTTCACCTTAT
TTACCATTTGGTGGTGGTAGACATAGATGTATTGGGGAACAATTTGCTTATGTTCAATTG
GGAACCATTTTAACTACTTTTGTTTATAACTTAAGATGGACTATTGATGGTTATAAAGTG
CCTGACCCTGATTATAGTTCAATGGTGGTTTTACCTACTGAACCAGCAGAAATCATTTGG
GAAAAAAGAGAAACTTGTATGTTTTAA

Supplementary figure 1

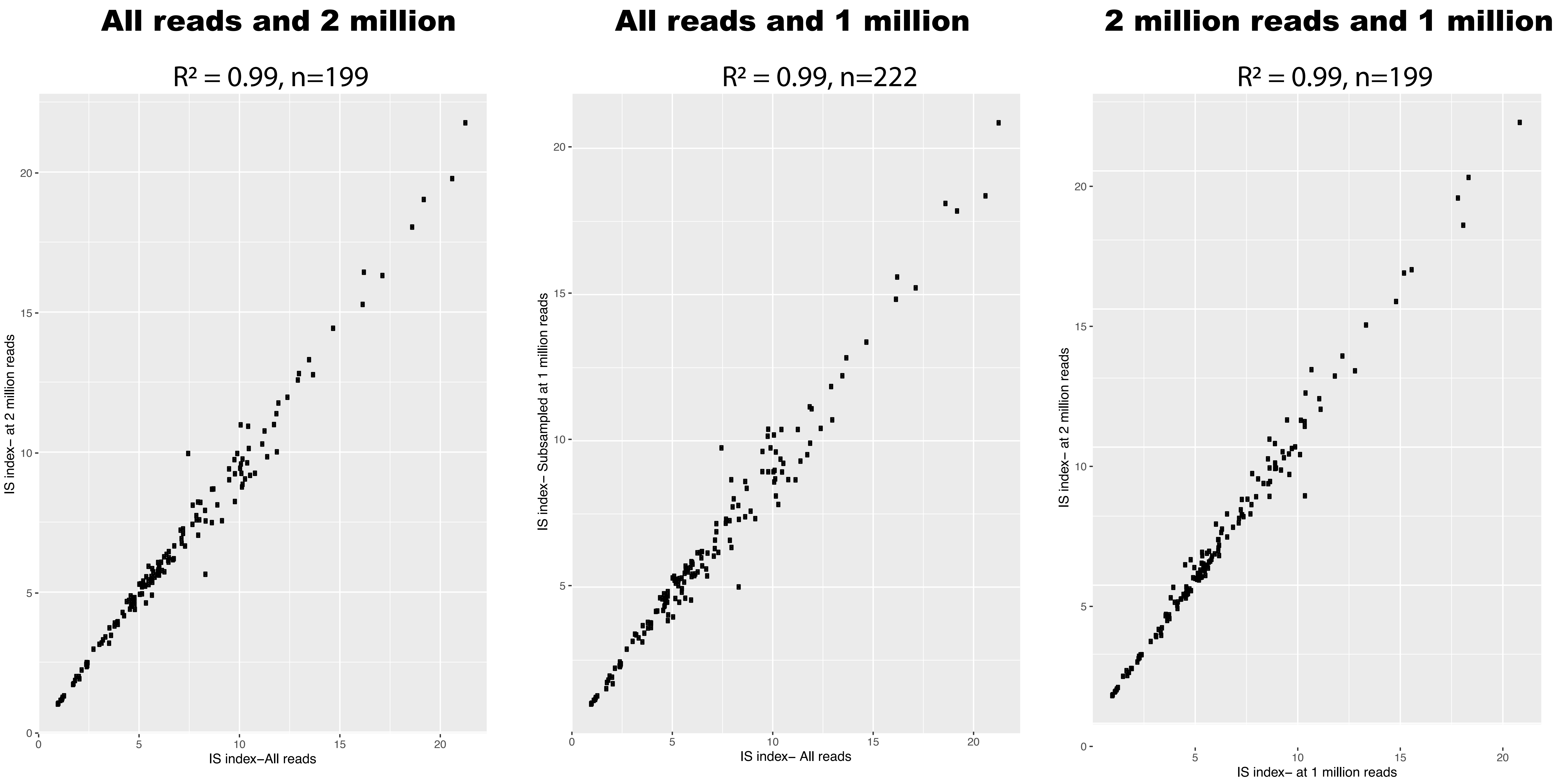

Supplementary figure 1: Inverse simpson (IS) index calculated for each sample compared between reads subsampled to 1 million and 2 million reads. Inputs were species relative abundance calculated from Metaphlan2. Correlation coefficients and number of samples in the analyses are shown.

Supplementary figure 2.2

a)

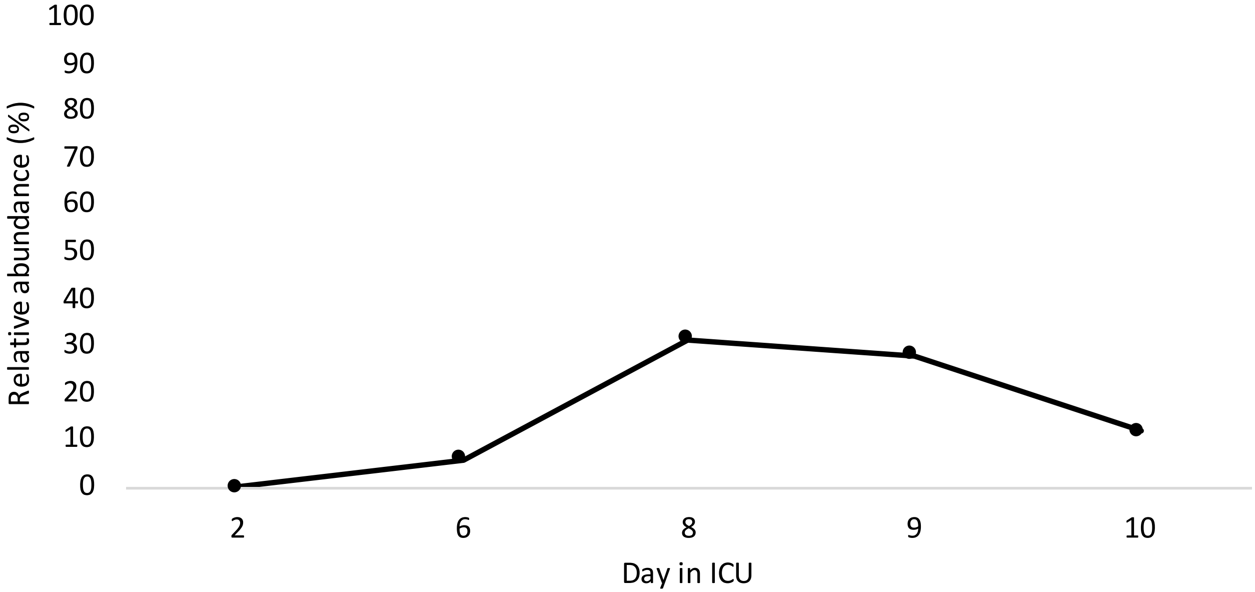

b)

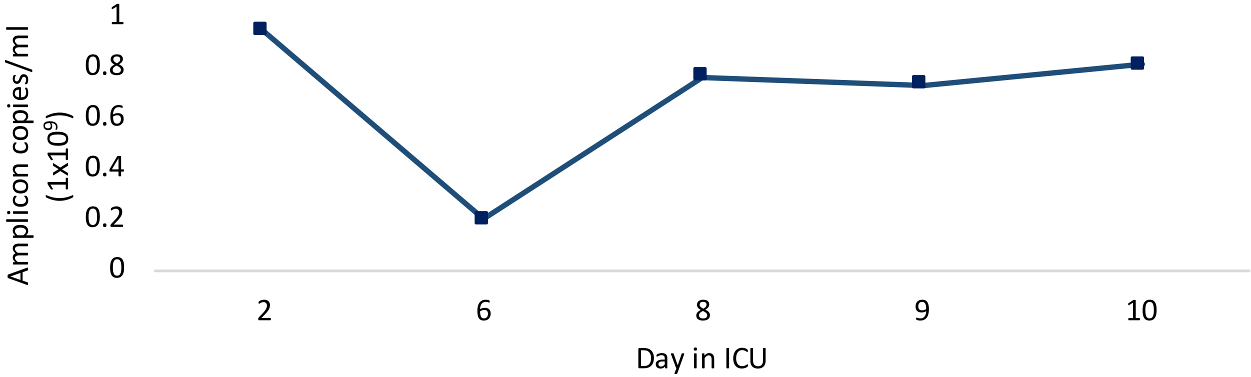

Supplementary figure 2.2

a) Patient 10: Relative abundance of *Methanobrevibacter* from Metaphlan2

b) Patient 10: Amplicon copies of 16S rRNA from qPCR

Supplementary figure 2.2

a)

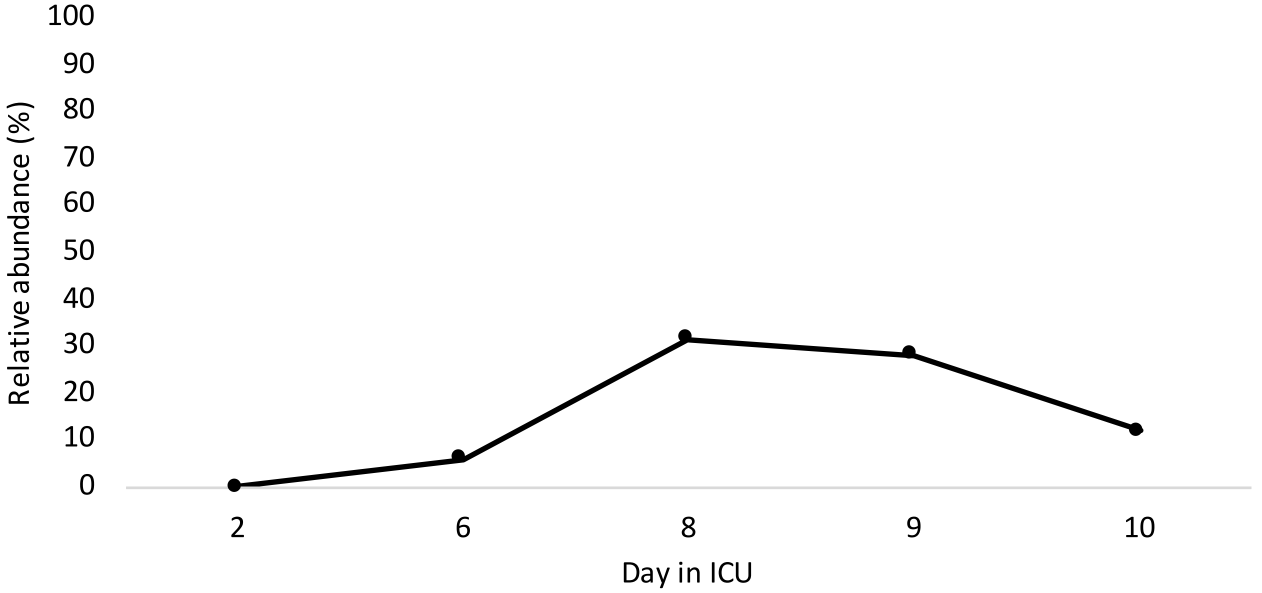

b)

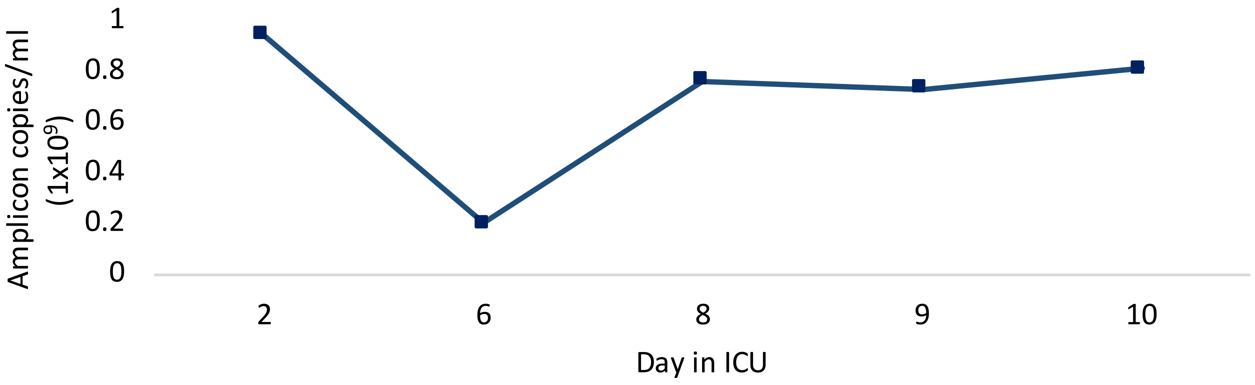

Supplementary figure 2.2

a) Patient 10: Relative abundance of *Methanobrevibacter* from Metaphlan2

b) Patient 10: Amplicon copies of 16S rRNA from qPCR

Supplementary figure 2.3

a)

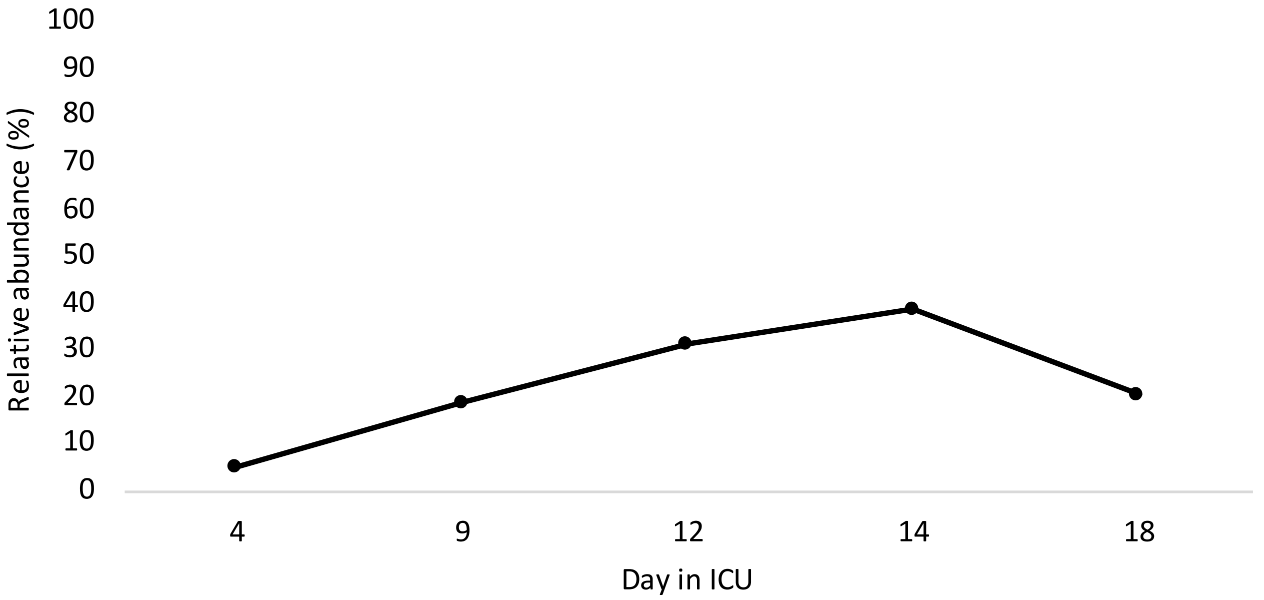

b)

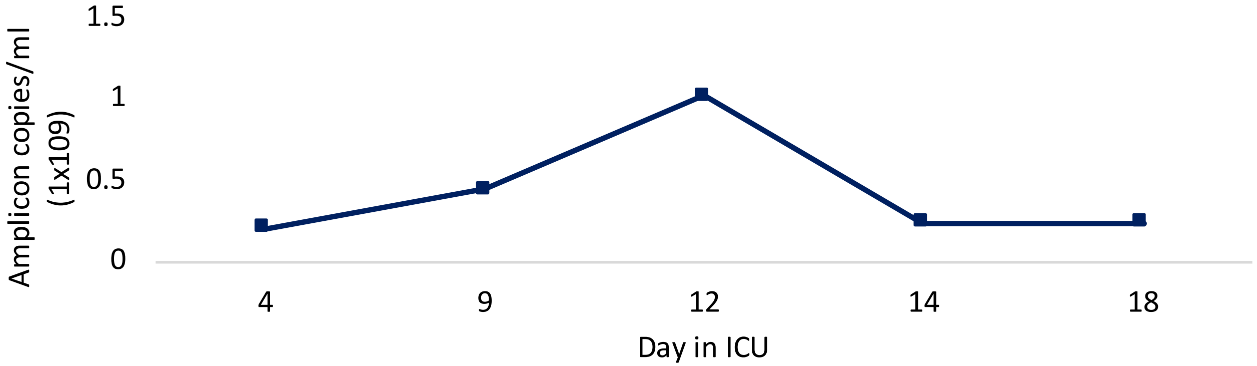

Supplementary figure 2.3

a) Patient 57: Relative abundance of *Methanobrevibacter* from Metaphlan2

b) Patient 57: Amplicon copies of 16S rRNA from qPCR

Supplementary Figure 3

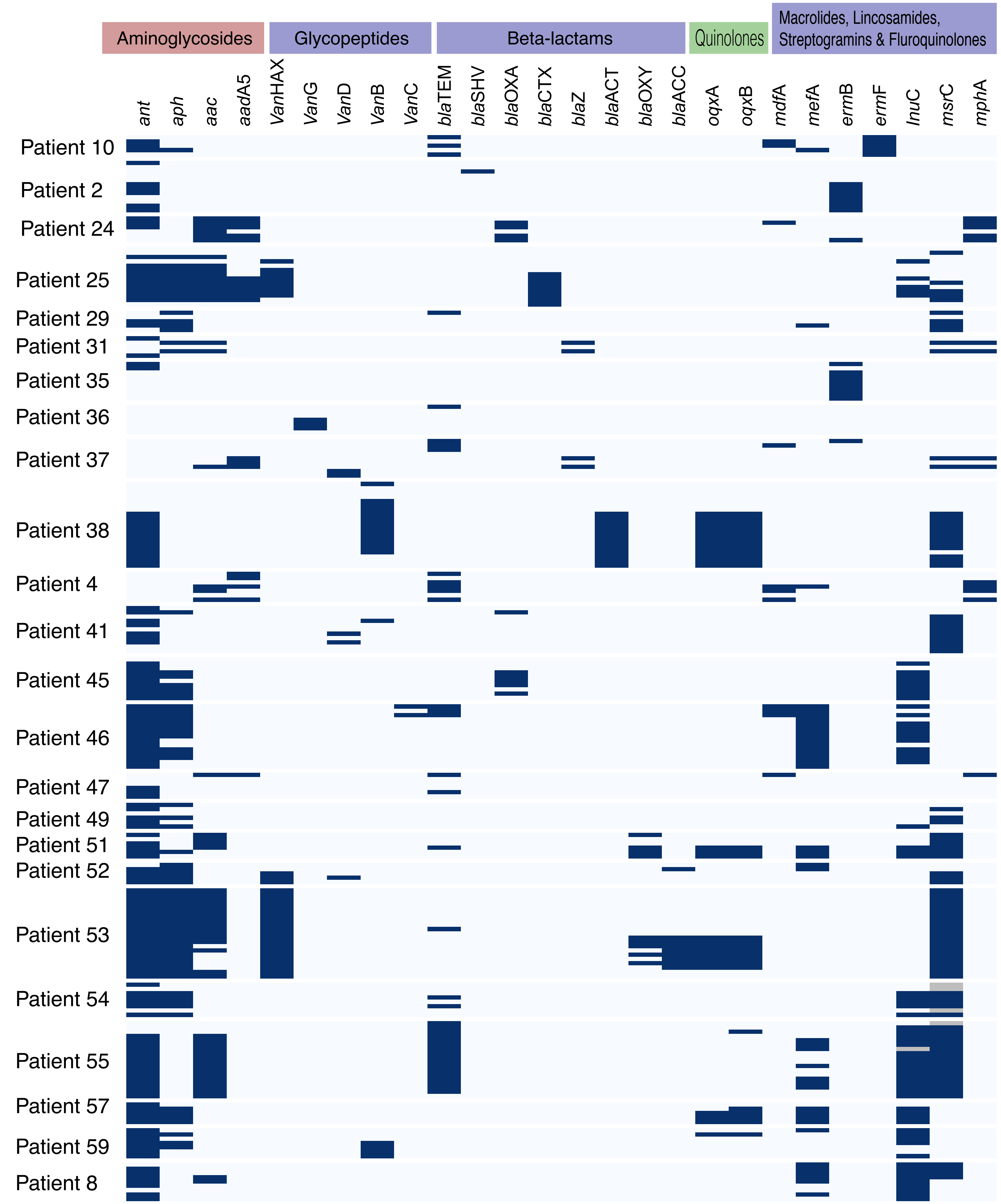

Supplementary figure : Presence or absence of antibiotic resistance genes in individual samples from all patients. Blue represents presence of an antibiotic resistance gene.

Supplementary figure 4

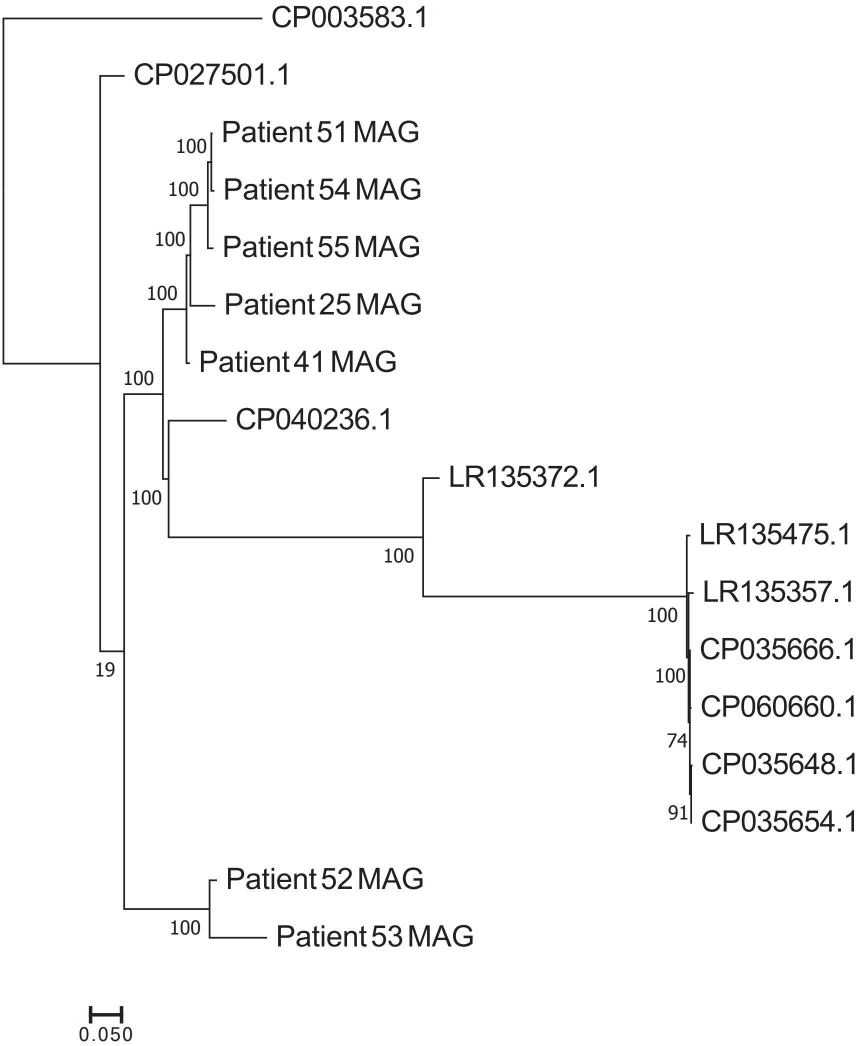

Supplementary figure 4: Phylogenetic tree based on the core-genome SNP of the MAGS and genomes.
The tree was constructed using RaxML with 100 rapid bootstrap analyses. Seven MAGs and 10 publicly available genomes were used. The tree was rooted with *E. faecium* DO (CP003583.1)

Supplementary figure 5.1

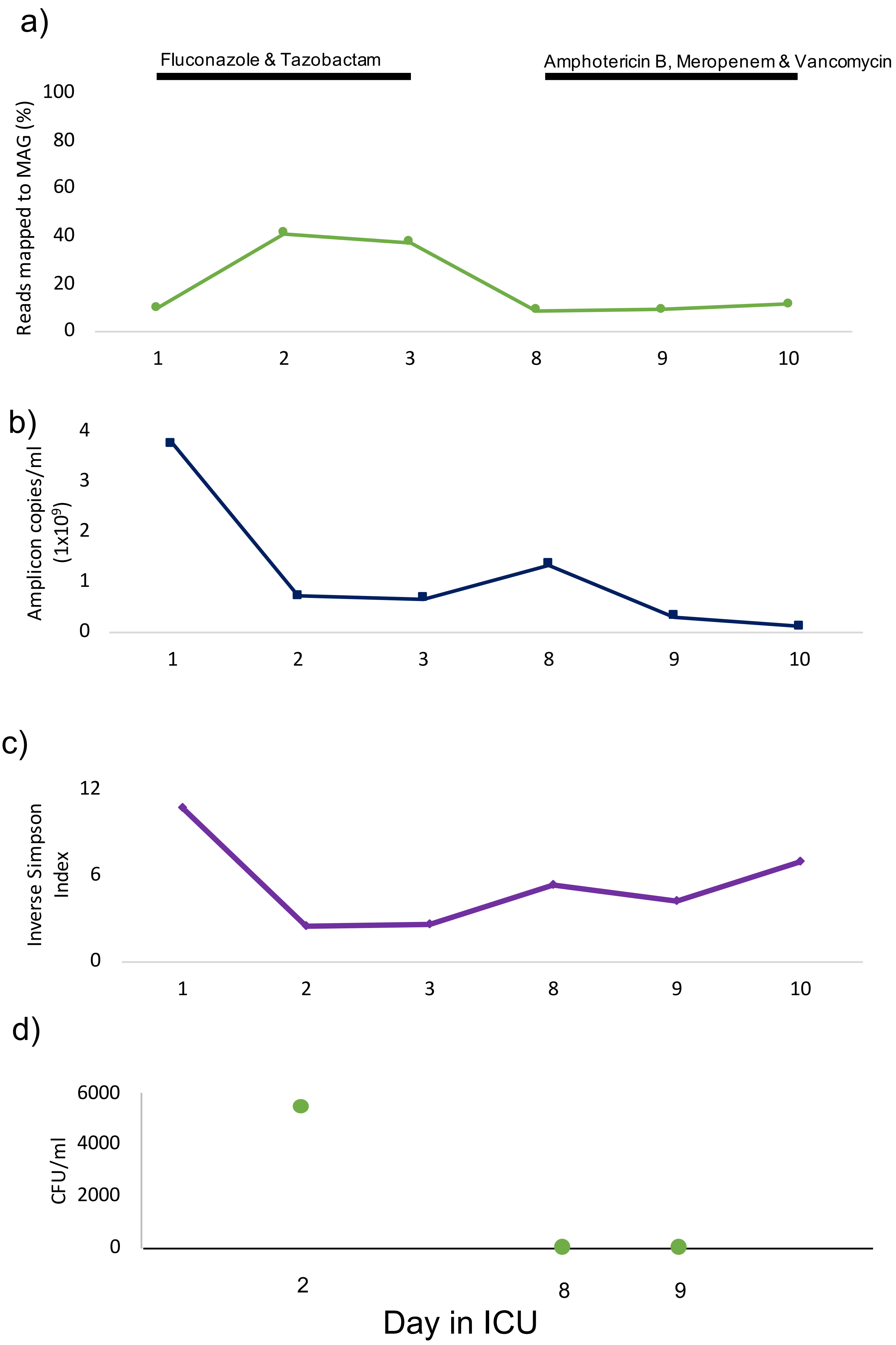

Supplementary figure 5.1
Patient 24

a) Percentage of reads from each sample mapped to *E. coli* MAG
b) Amplicon copies of 16S rRNA from qPCR
c) Inverse Simpson index calculated from Metaphlan2
d) Colony forming units counted for *E. coli* in selected samples

Supplementary figure 5.2

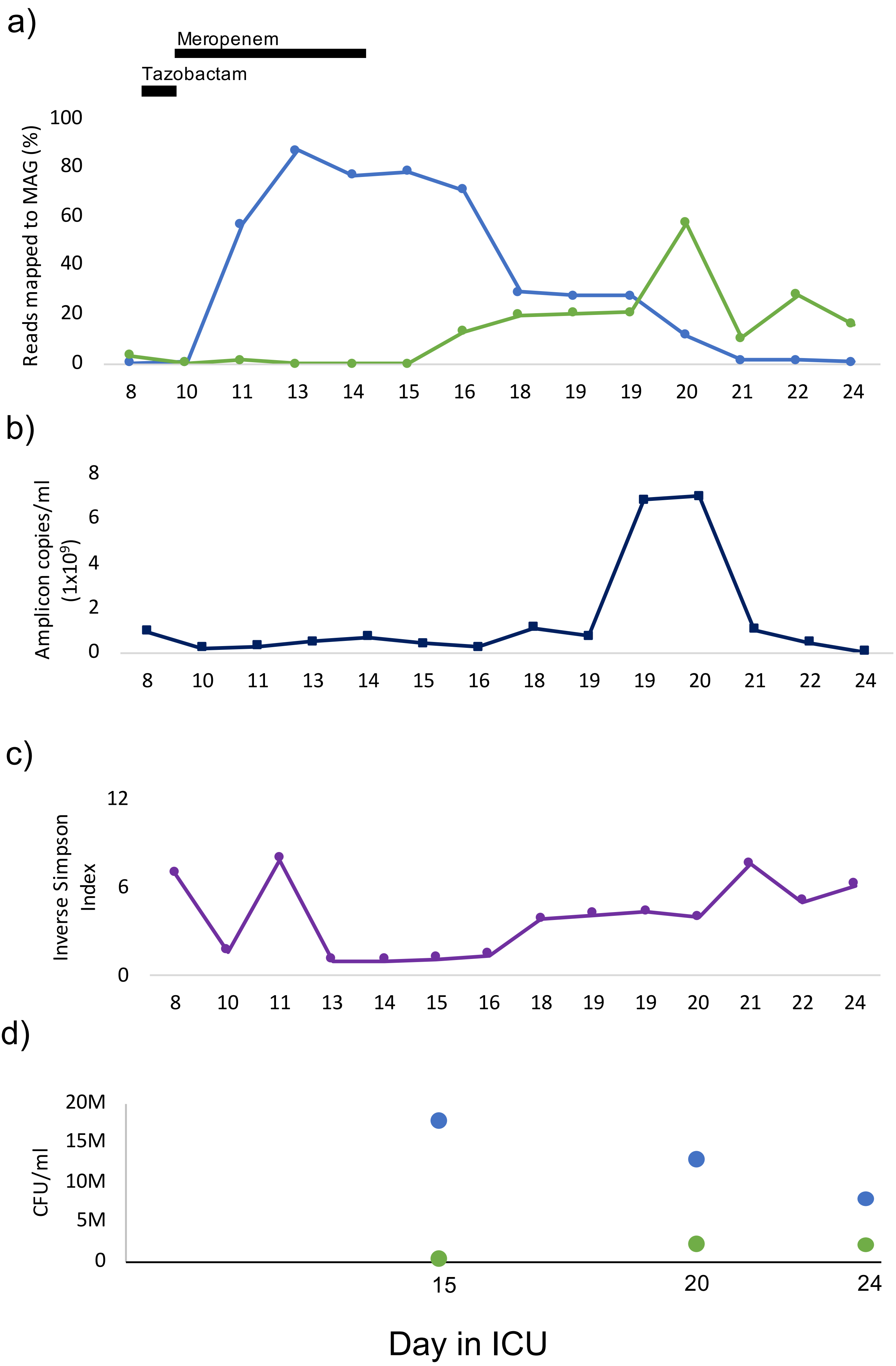

Patient 25

a) Percentage of reads from each sample mapped to *E. faecium* MAG (Blue) and *E. coli* MAG (green)
b) Amplicon copies of 16S rRNA from qPCR
c) Inverse Simpson index calculated from Metaphlan2
d) Colony forming units counted for *E. faecium* (blue) and *E. coli* (green) for selected samples

Supplementary figure 5.3

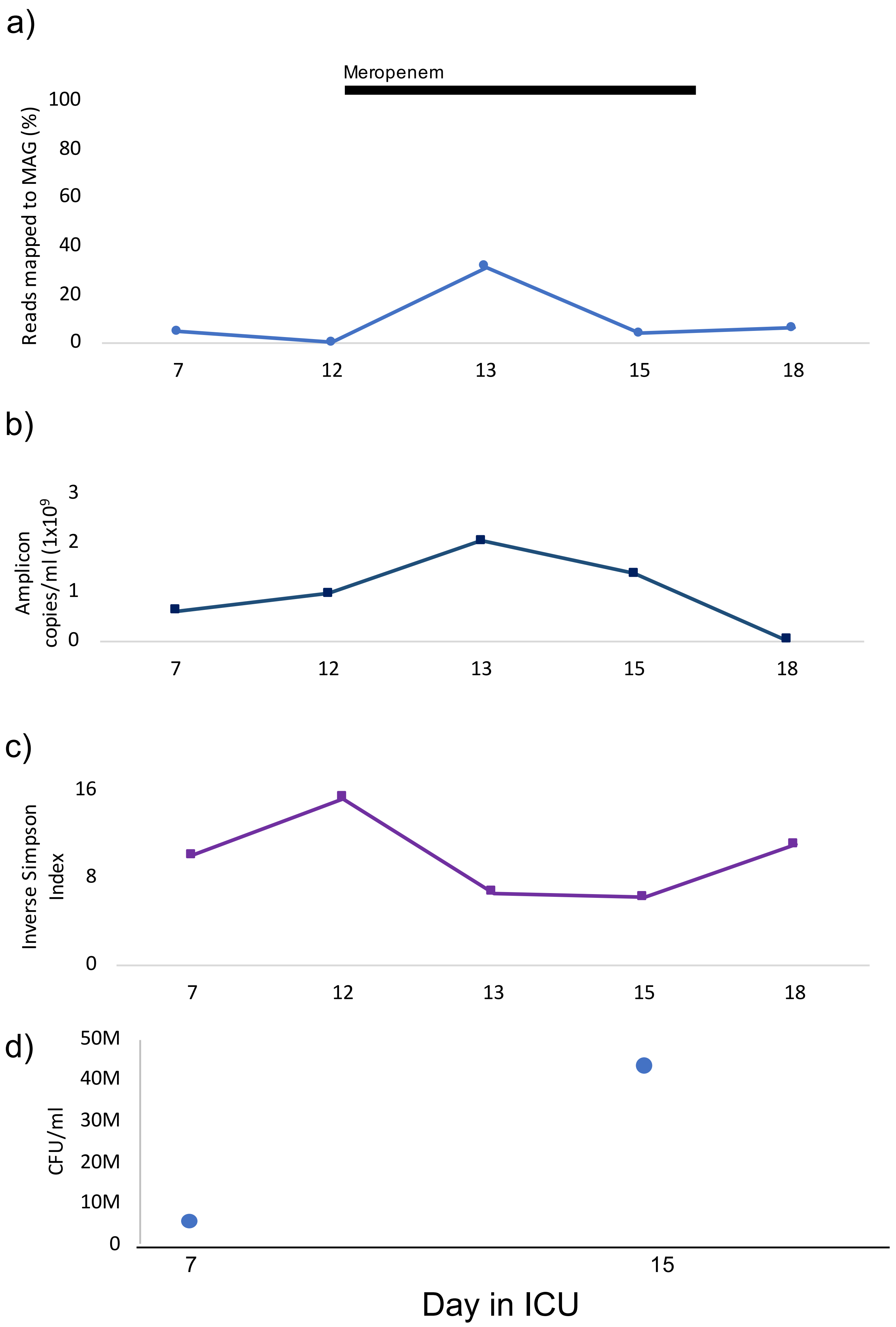

Patient 29

a) Percentage of reads from each sample mapped to *E. faecium*
b) Amplicon copies of 16S rRNA from qPCR
c) Inverse Simpson index calculated from Metaphlan2
d) Colony forming units counted for *E. faecium*

Supplementary figure 5.4

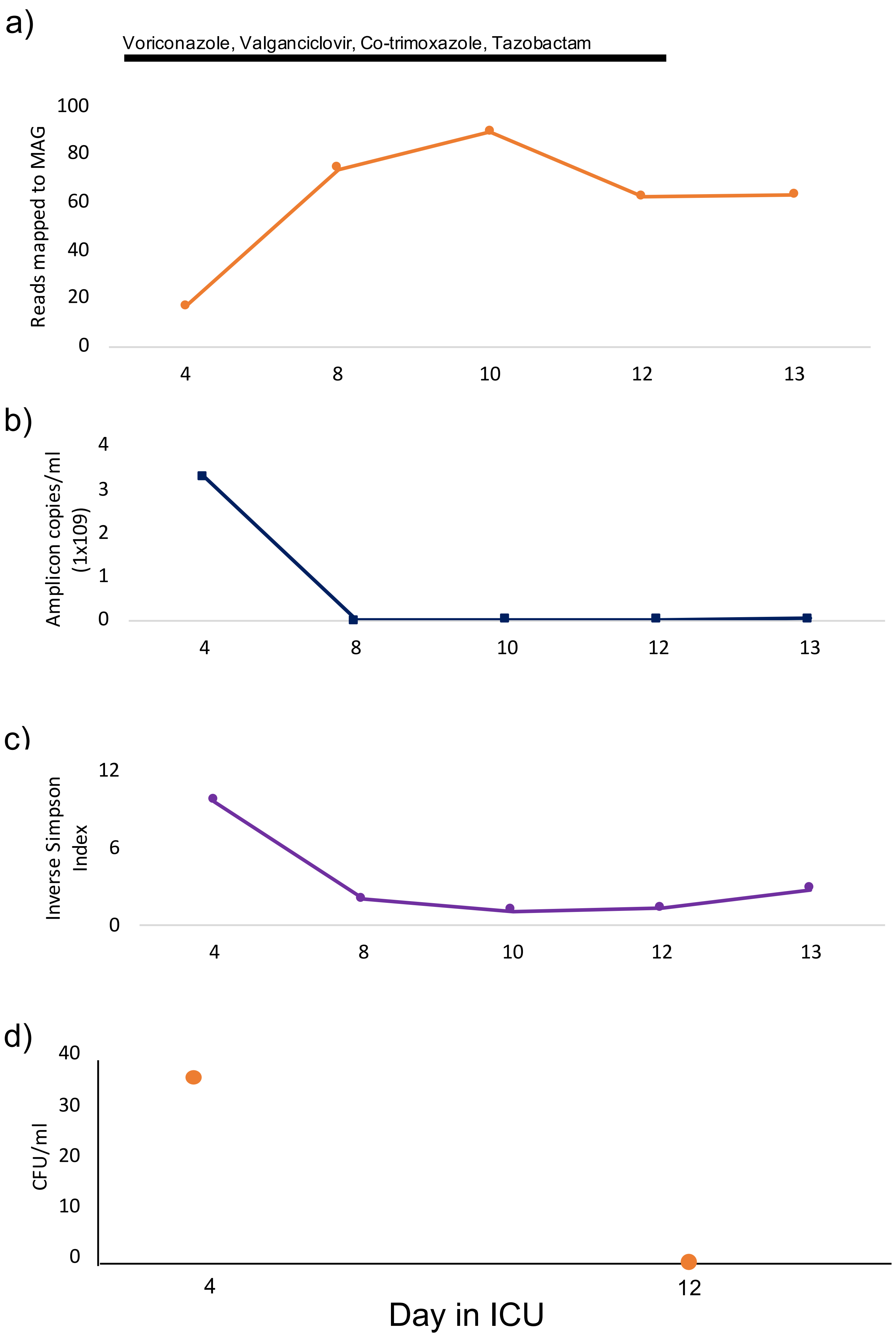

Patient 35

a) Percentage of reads from each sample mapped to *C. albicans*
b) Amplicon copies of 16S rRNA from qPCR
c) Inverse Simpson index calculated from Metaphlan2
d) Colony forming units counted for *C. albicans*

Supplementary figure 5.5

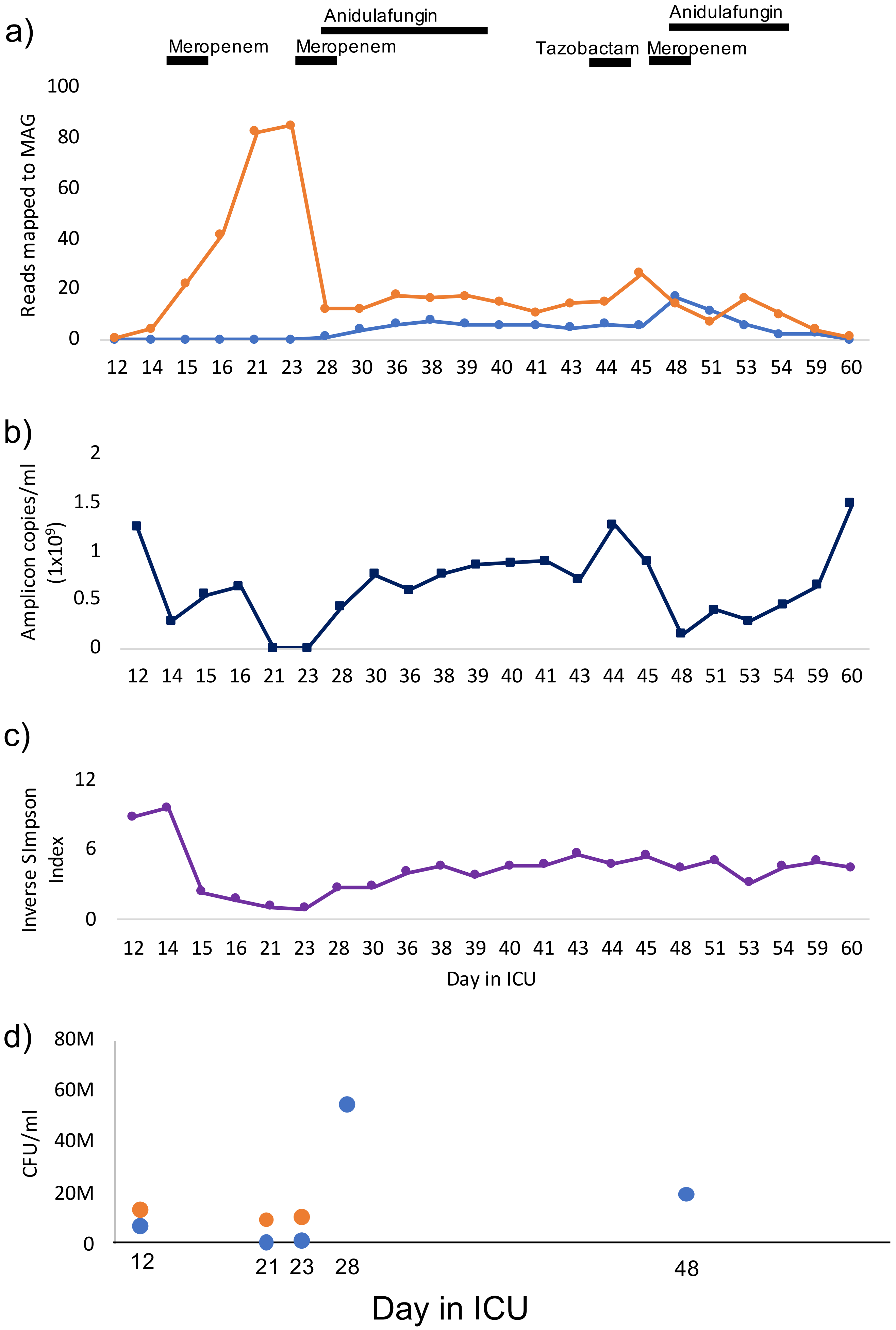

SPatient 38

a) Percentage of reads from each sample mapped to *C. albicans* (orange) and *E. faecium* (blue)
b) Amplicon copies of 16S rRNA from qPCR
c) Inverse Simpson index calculated from Metaphlan2
d) Colony forming units counted for *C. albicans* (orange) and *E. faecium* (blue)

Supplementary figure 5.6

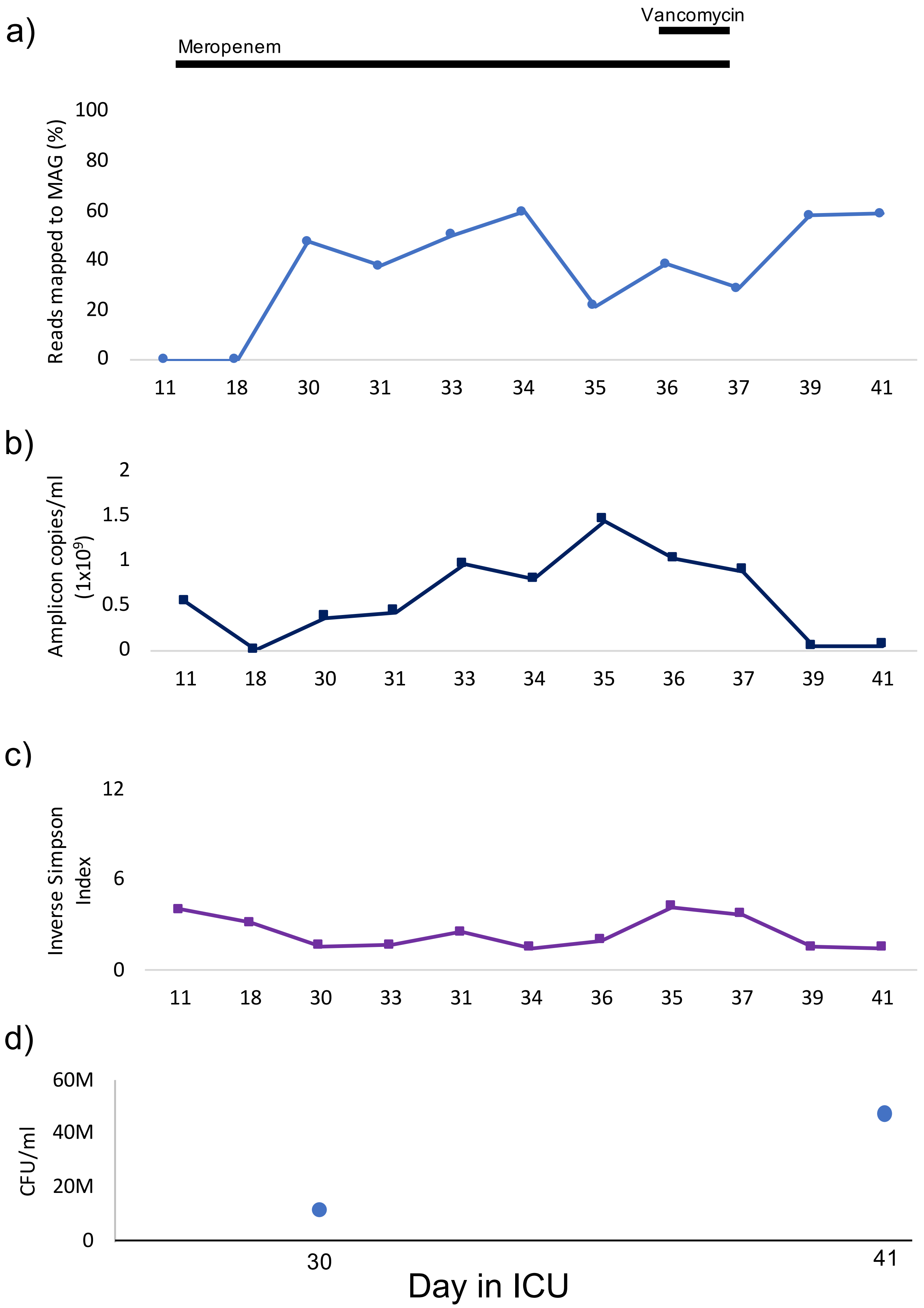

Patient 41

a) Percentage of reads from each sample mapped to *E. faecium*
b) Amplicon copies of 16S rRNA from qPCR
c) Inverse Simpson index calculated from Metaphlan2
d) Colony forming units counted for *E. faecium*

Supplementary figure 5.7

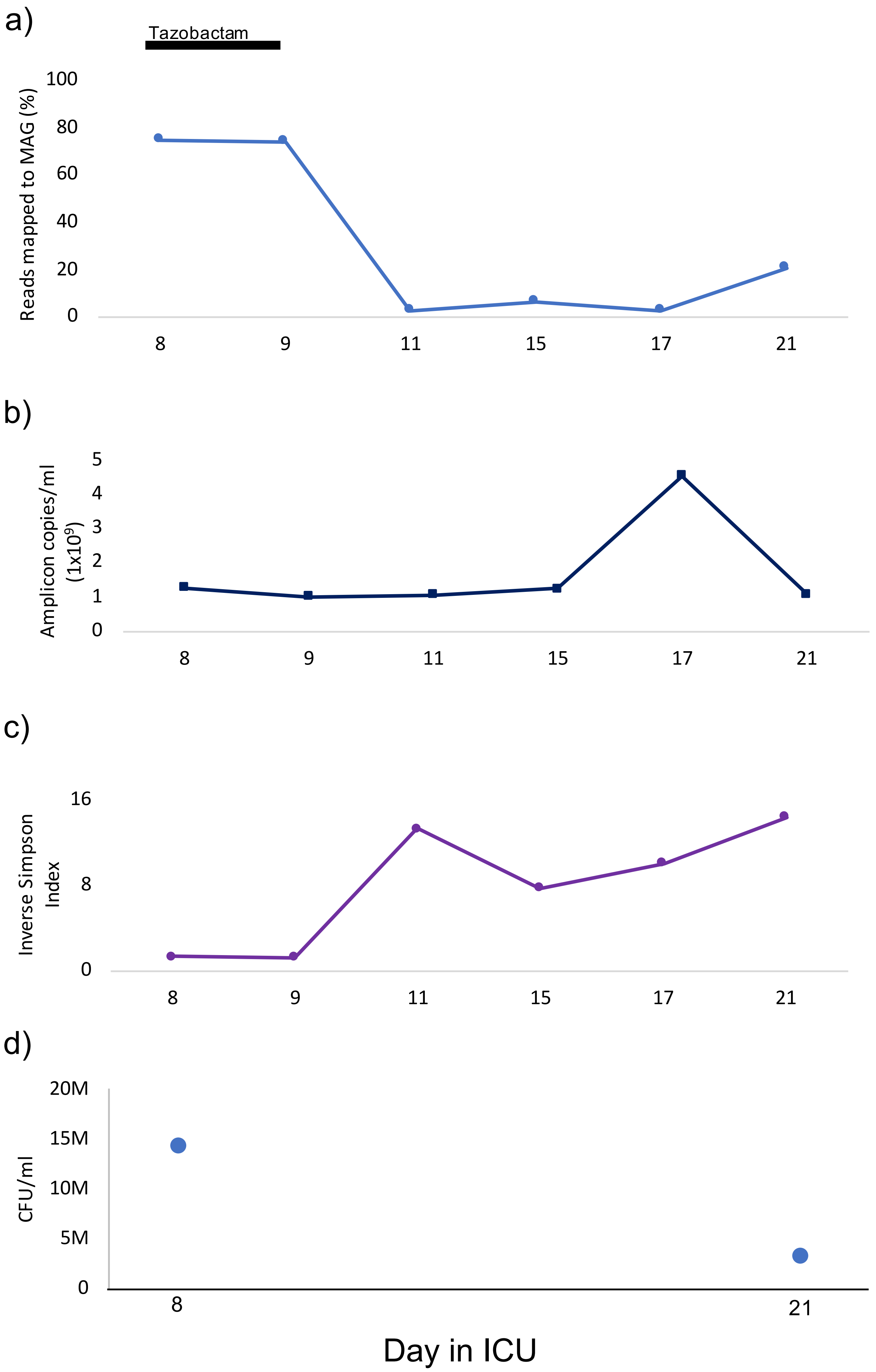

Patient 51

a) Percentage of reads from each sample mapped to *E. faecium*
b) Amplicon copies of 16S rRNA from qPCR
c) Inverse Simpson index calculated from Metaphlan2
d) Colony forming units counted for *E. faecium*

Supplementary figure 5.8

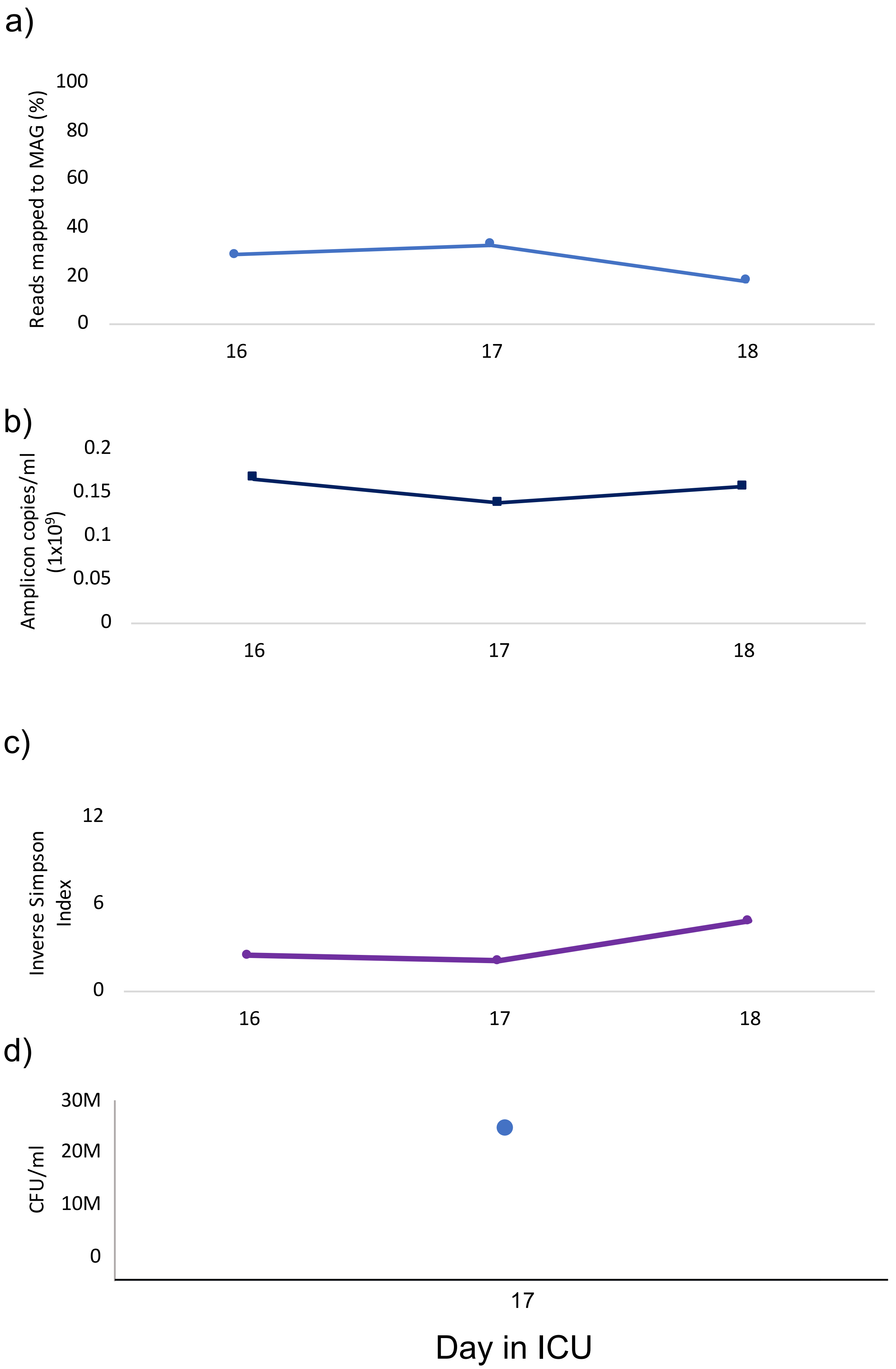

Patient 52

a) Percentage of reads from each sample mapped to *E. faecium*
b) Amplicon copies of 16S rRNA from qPCR
c) Inverse Simpson index calculated from Metaphlan2
d) Colony forming units counted for *E. faecium*

Supplementary figure 5.9

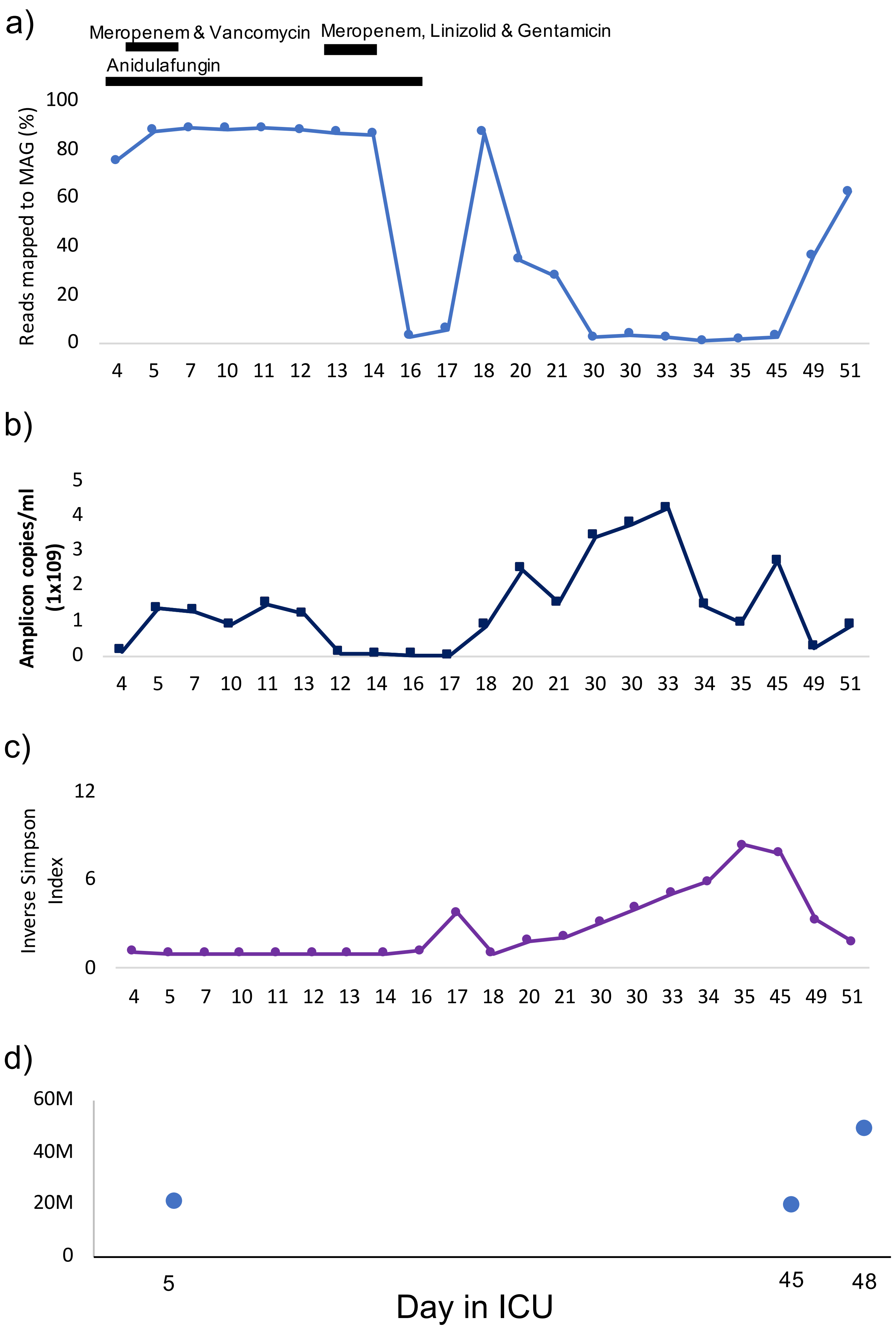

Patient 53

a) Percentage of reads from each sample mapped to *E. faecium*
b) Amplicon copies of 16S rRNA from qPCR
c) Inverse Simpson index calculated from Metaphlan2
d) Colony forming units counted for *E. faecium*

Supplementary figure 5.10

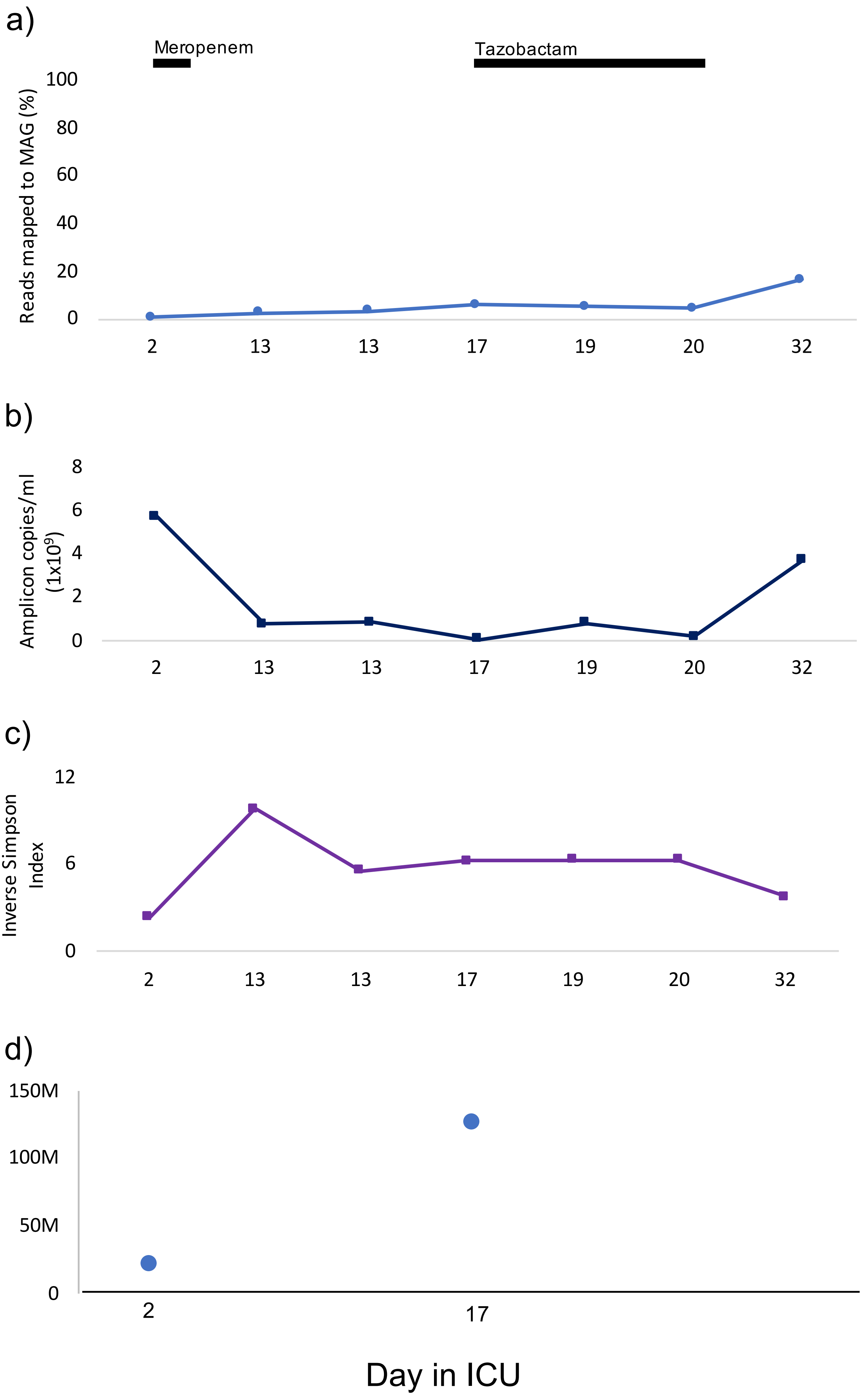

Patient 54

a) Percentage of reads from each sample mapped to *E. faecium*
b) Amplicon copies of 16S rRNA from qPCR
c) Inverse Simpson index calculated from Metaphlan2
d) Colony forming units counted for *E. faecium*

Supplementary figure 5.11

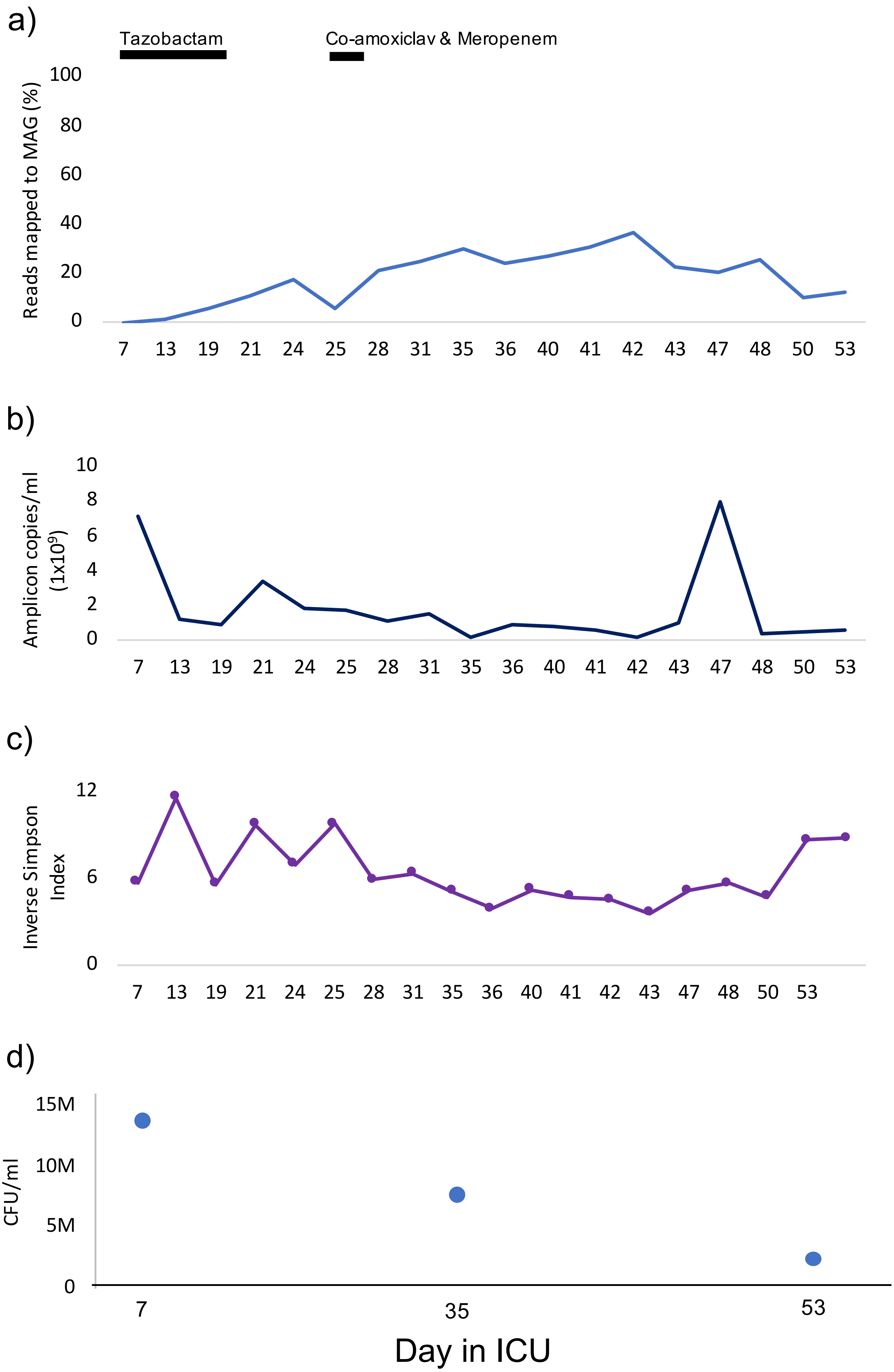

Patient 55

a) Percentage of reads from each sample mapped to *E. faecium*
b) Amplicon copies of 16S rRNA from qPCR
c) Inverse Simpson index calculated from Metaphlan2
d) Colony forming units counted for *E. faecium*
